# Supplementary material for: Porous functionalized polymers enable generating and transporting hyperpolarized mixtures of metabolites
Source: Nat Commun. 2021 Aug 4;12:4695. doi: 10.1038/s41467-021-24279-2 (PMC8338986; doi:10.1038/s41467-021-24279-2)
Supplement: Supplementary file 1 — Supplementary information. [file 41467_2021_24279_MOESM1_ESM.pdf]

Supplementary Information

Porous Functionalized Polymers enable Generating and Transporting Hyperpolarized Arbitrary Solutions.

Théo El Daraï,[1,2] Samuel F. Cousin,[1] Quentin Stern,[1] Morgan Ceillier,[1] James G. Kempf,[5] Dmitry Eshchenko,[3] Roberto Melzi,[6] Marc Schnell,[3] Laurent Gremillard,[4] Aurélien Bornet,[1] Jonas Milani,[1] Basile Vuichoud,[1] Olivier Cala,[1] Damien Montarnal,*[2] and Sami Jannin[1]*

[1] *Univ. Lyon, Centre de RMN à Très Hauts Champs de Lyon, FRE2034 - CNRS/UCBL/ENS de Lyon, 5 rue de la Doua, 69100 Villeurbanne, France.*
[2] *Univ Lyon, CPE Lyon, CNRS, Catalyse, Chimie, Polymères et Procédés, UMR 5265, F-69003, Lyon, France*
[3] *Bruker Biospin, 8117 Fallanden, Switzerland*
[4] *Univ Lyon, INSA Lyon, MATEIS UMR CNRS 5510, Bât. Blaise Pascal, 7 Avenue Jean Capelle, Villeurbanne, France*
[5] *Bruker Biospin, 15 Fortune Dr., Billerica, Massachusetts 01821, United States*
[6] *Bruker Italia Srl, 20158 Milano, Italy*

*damien.montarnal@univ-lyon1.fr, samuel.cousin89@gmail.com

Table of contents

1 Supplementary Methods 3
1.1 Materials 3
1.2 SEM characterization 3
1.3 Characterization of porosity 3
1.3.1 Mercury intrusion porosimetry 3
1.3.2 Nitrogen physisorption 3
1.4 Rheology 3
1.5 EPR Quantification 4
1.6 Polarizer apparatus 4
1.7 ^{1}H DNP experiments 4
1.8 ^{13}C DNP experiments 5
1.9 Dissolution, transfer and injection experiments 5
1.10 Hyperpolarized liquid-state NMR measurements 5
1.11 HYPOPs Synthesis and Preparation 5
1.12 HYPOP washing and drying 6
1.13 Methods of impregnation 6
1.14 Post dissolution filtration system 6
1.15 Method for polarization quantification in solid state 7
1.16 ^{13}C relaxation measurement 7
1.17 Calculation of enhancements after dissolution 8
1.18 HYPOP-I composition 9

2 Supplementary Discussion 10
2.1 SEM analyses of porous samples with varying compositions 10
2.2 SEM analyses of HYPOP-I samples 12
2.3 Monitoring of the curing by rheology 15
2.4 Mercury Intrusion Porosimetry 15
2.5 Nitrogen physisorption 17
2.6 EPR Calibration 18
2.7 EPR spectra and quantification of radicals 19
2.8 Thermal equilibrium & DNP buildup 19
2.9 Cross-polarization 20
2.10 Swelling measurements 20
2.11 Microwaves optimization at 1.2K and 7.05 T, on HYPOP-I: 22
2.12 Dry HYPOP ^{1}H DNP build-ups 22
2.13 Impregnated (10%$_v$ H_2O / 10%$_v$ ETOD$_6$ / 80%$_v$ H_2O) HYPOP ^{1}H DNP build-ups 28
3 Supplementary References 32

1 Supplementary Methods

1.1 Materials

(2,2,6,6-Tetramethylpiperidin-1-yl)oxy (TEMPO), used as reference for EPR calibration was bought from Sigma-Aldrich.

4-Amino-2,2,6,6-tetramethylpiperidine-1-oxyl (amino TEMPO), used for syntheses was bought from Tokyo Chemical Industry.

Potassium Bromide (KBr) used as passive media for EPR calibration was bought from ACROS.

Diglycidyl Ether of Bisphenol A (DGEBA), used for syntheses was bought from Sigma-Aldrich.

Isophorone Diamine (IPDA), used for syntheses was bought from Tokyo Chemical Industry.

Polypropylene glycol (PPG *x* with x the average molar mass in number), used for synthesis was bought from Alfa Aesar/ Fisher (400 g/mol), Sigma Aldrich (725, 1000 & 4000 g/mol), Tokyo Chemical Industry (192 g/mol).

^{13}C-tagged Acetate, Urea, Glycine, formate and pyruvate were bought from Sigma-Aldrich.

1.2 SEM characterization

Scanning Electron Microscopy experiments were performed in the "Centre Technologique des Microstructures (CTμ)" in Lyon, on a ZEISS Merlin Compact after deposition of 10 nm of copper using a BAL-TEC Med 20 coating system.

1.3 Characterization of porosity

1.3.1 Mercury intrusion porosimetry

Mercury intrusion porosimetry was performed on a AutoPore IV 9400 apparatus from Micromeritics. About 100 mg of samples was degassed in the porosimeter to less than 50 μm Hg before the mercury intrusion. The intrusion was performed in the pressure range of 0.035 to 4000 bar, allowing the penetration of the pores of diameter ranging between 3 nm and 350 μm, with an accuracy of about 0.25%. Based on the assumption of cylindrical pores, the apparent pore size distribution was calculated by the Washburn equation: $D = (-4\sigma\cos\Theta)/P$, where P is the absolute injection pressure (Pa), D is the pore access diameter (m) when mercury enters at the pressure P, Θ is the contact angle between mercury and the pore surface (assumed to be 130° in the experiments) and σ is the interfacial tension of mercury (set to 0.485 $J.m^{-2}$).The apparent total porous volume is calculated by cumulating the incremental pore volumes between 0.65 bar and 4000 bar (which corresponds to pore diameters below 20 μm).

1.3.2 Nitrogen physisorption

Nitrogen physisorption isotherms were performed using Nitrogen at 77 K (liquid nitrogen) on an ASAP 2020 apparatus from Micromeritics. Prior to analyses, the samples (about 100 mg) were degassed at 40°C for at least 3 h. Adsorption and desorption isotherms were run in a range of relative pressures from P/P0= 0.05 to 0.98

1.4 Rheology

In situ monitoring of the curing reaction was carried out using a HAAKE MARS 60 rheometer from Thermo Fisher using 60 mm plan geometries and gap about 250 μm. Initially, the low-viscosity fluid was characterized at constant shear rate ($\dot{\gamma}$=10 s^{-1}) until the viscosity increased above 0.2 Pa.s. Subsequently, oscillatory shear (ω=10 $rad.s^{-1}$) at constant stress (τ=1 Pa) were carried out to monitor the gelation.

1.5 EPR Quantification

Electron paramagnetic resonance (EPR) experiments were performed on a continuous wave X band EMXnano apparatus from Bruker. Microwave source is working at 9.63 GHz and experiments were performed at 343 mT with 40 mT of sweeping. Analyses were performed using 4 mm quartz tubes bought from Wildman. Data processing and especially baseline correction were performed manually on Matlab, using polynomials fits.

Powders were prepared by crushing the polymers with a lancet, and sorted with a range of sieves (1 mm / 500 µm / 250 µm thresholds). Intermediate fractions (1 mm > d > 500 µm, and 500 µm > d > 250 µm) were kept and used for analyses and DNP experiments. The powders were packed into 4 mm quartz tubes (20 mm height, about 20 mg) and analyzed with EPR. The corresponding spectra were integrated a first time before baseline correction. After that, the spectra were integrated a second time and corrected using the following formula:[1]

$$I_{corrected} = \frac{\iint_{325}^{355} S(B_0)\mathrm{d}B_0}{Q_{factor} \times B \times \sqrt{P} \times NS \times 10^{\frac{20}{RG}} \times T_c} \qquad \text{(Supplementary Equation 1)}$$

with:

- Q_{factor} Quality factor of the cavity under experimental conditions.
- B_0 Magnetic field (mT).
- B Field modulation (Gauss).
- P Microwaves power (mW).
- T_c Conversion time (ms).
- NS Number of scans.
- RG Receiver gain (dB).

NS and *RG* were both directly taken into account by the software, and T_c was kept constant for all experiments (5 ms).

1.6 Polarizer apparatus

All DNP measurements were performed with a prototype dDNP Polarizer developed by *Bruker Biospin*, with a helium bath cryostat operating between 1.2 K<T<4.2 K at a magnetic field of 7.05 T and equipped with $^1H/^{13}C$ homemade NMR probe. Microwaves were generated with a Virginia Diode system (8-20 GHz VDI synthesizer with a 198 GHz AMC amplifier / multiplier chain). For the characterization of HYPOP, a KelF (Polychlorotrifluoroethylene) sample holder was used to reduce the ^1H signal background. For dissolution experiments, a more robust PEEK sample holder was used.

1.7 ^1H DNP experiments

HYPOPs where ground, and a size between 250 and 500 µm was selected using sieves. Prior to DNP experiments, HYPOP powders were used as is or impregnated with solutions to be hyperpolarized, placed in the sample cup, and inserted in the liquid helium bath of the dDNP polarizer at 4.2 K. ^1H NMR signals were measured at 3.8 K (700 mBar pressure), with 0.1° pulses every 5 seconds during 10 min until reaching the thermal equilibrium plateau. The quality factor of the ^1H NMR circuit was by attenuated by adding g a 50-Ohm resistor in series with the tuning and matching box, so as to decrease radiation-damping (potentially very intense at high polarization values) which can lead to underestimation of the polarized ^1H signal. ^1H polarization curves were recorded with at 1.4 K and 7.05 T, with the following microwave parameters: frequency of f_{uw}=197.648 GHz, triangular frequency modulation of width Δf_{uw} = 160 MHz, modulation rate f_{mod} = 500 Hz, and estimated power in the sample cavity P_{uw} = 30 mW.

1.8 ^{13}C DNP experiments

After inserting the sample, ^{13}C NMR signals were measured with 5° pulse every 30 min during 6 h at 3.8 K and once the plateau was reached, the thermal equilibrium NMR signal was recorded with a series of 64 pulses for improved sensitivity (See section 8.4 in the SI). The same procedure was previously applied for the measurement of the ^{13}C background signal (without sample). ^{1}H→^{13}C CP was performed using a 6 ms contact every 5 min to allow time for ^{1}H polarization to build-up and diffuse in the frozen solution in the pores of HYPOP. The CP matching condition was realized with 23 kHz B_1 field on both ^{1}H and ^{13}C channels (with 8 ms square pulse of ^{1}H (8 W) and 50.100 ramps on ^{13}C (150 W)). Adiabatic half passage pulses (100 kHz, 175 µs with 12 W for the ^{1}H channel and 150 W for the ^{13}C channel) were used to flip the ^{1}H and ^{13}C magnetization in the transverse plane before CP contact and to restore magnetization along z afterwards.

1.9 Dissolution, transfer and injection experiments

To ensure the that the fluid path does not cross a zero field, a solenoid of 1.5m length has been built around the capillary, and fed with a current of 2 A, thus generating a 4 mT all along the transfer except for the filtering system placed in close proximity to the benchtop spectrometer. 7mL of D_2O solvent where pressurized at 6 Bar and heated to reach 9 Bar. After the dissolution, the HYPOP-**I** matrices mixed together with the molecules of interest are pushed with hot D_2O through a filtering device described in section 6 in the SI. Finally, the solution was fed into a Bruker's Prototype NMR injector directly placed in the Fourier80 benchtop NMR spectrometer.

1.10 Hyperpolarized liquid-state NMR measurements

Liquid-state hyperpolarized NMR spectra were measured in a Fourier 80 MHz *Bruker Biospin* benchtop spectrometer (^{1}H frequency 80.222 MHz and ^{13}C frequency 20.1718 MHz). After injection, ^{13}C signals were recorded every 5 s with a 5° nutation angle pulse. Final hyperpolarization enhancement was calculated by cross-calibration with a highly concentrated 3 M [1-^{13}C] sodium acetate reference sample, with a simple method described in detailed in section 8.5 in the SI.

1.11 HYPOPs Synthesis and Preparation

The synthesis of HYPOP samples is performed by weighting aminoTEMPO in a round bottom 14 mL polypropylene tube. As amino TEMPO is highly hygroscopic, this procedure is carried out in a glovebox. Quickly after removing the tube from the glovebox are added DGEBA, IPDA and PPG (See Supplementary Table). After mild heating of the mixture to decrease viscosity, it is thoroughly degassed using high vacuum and intense stirring. When the sample is fully homogeneous, transparent and bubble-free the tube is put in a dry bath heater (Corning LSE) and cured at 102°C for 24h.

After the curing reaction, the post-polymerization process consists in i) trimming edges where a skin layer has formed, ii) washing in large amounts of ethanol (3 times) and DI-water (3 times). After the last washing step with water, the wet samples are frozen in liquid nitrogen and freeze-dried (Freezone 4.5 from Labconco, P=0.01 mbar, $T_{collector}$=-104°C). Dry HYPOPs are manually crushed then sifted before being analyzed with EPR or used in dDNP experiments. HYPOP are stored at room temperature and atmospheric conditions in polypropylene tubes. The radical concentration remains stable for over a year in such conditions.

1.12 HYPOP washing and drying

Several polymers were synthetized with composition similar to HYPOP-I batch and compared before and after washing without trimming edges. Ratio of wet and dried masses showed an extraction rate of more than 95% with one case of extra losses during the freezing step of the freeze drying process (partial break of the polymer).

Radical concentrations were estimated by considering amino TEMPO masses and a 34% survival yield.

Synthesis	Washed mass (g)	Percentage of solvent (%)	Theoretical dry mass (g)	Obtained dry mass (g)	Extraction rate (%)	Estimated radical concentrations (mmol/g)
**1**	3,7855	84,7267	0,5782	0,6142	98,9	0
**2**	3,10399	85,2815	0,4569	0,5371	97,0	18
**3**	1,99611	84,9040	0,3013	0,3282	98,4	20
**4**	2,14777	84,7767	0,3270	0,3137	100,7	24
**5**	2,18059	84,9368	0,3285	0,3393	99,4	28

Supplementary Table 1*: Composition of HYPOP-I samples. The composition selected for HYPOP-A sample is highlighted.*

1.13 Methods of impregnation

Solutions were loaded inside HYPOP using the incipient wetness impregnation technic by pouring 3 times the HYPOP mass as solution for HYPOP containing less than 192 µmol g^{-1} of radical and 2 times if else.

1.14 Post dissolution filtration system

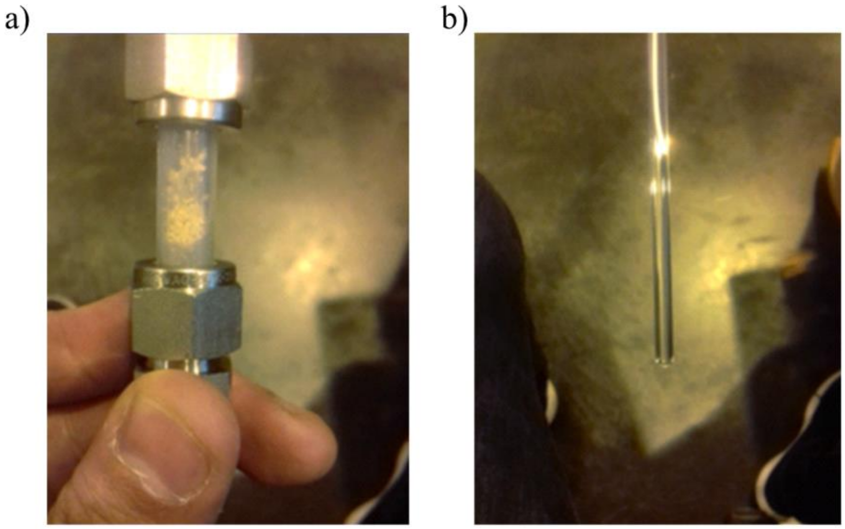

Supplementary Figure 1: *(a) In-line filter containing glass fibers, that retains HYPOP powder during the dissolution and transfer step. (b) final hyperpolarized solution transferred into a 5 mm NMR tube.*

1.15 Method for polarization quantification in solid state

Overall extent of polarization obtained in DNP were calculated by the following calculation:

$$P = P_{eq} \times \frac{I_{DNP}}{(I_{TE} - I_{Background})} \times \frac{G_{TE} \times NS_{TE} \times \theta_{TE}}{G_{DNP} \times NS_{DNP} \times \theta_{DNP}} \quad \text{(Supplementary Equation 2)}$$

with:

- TE Thermal Equilibrium (NMR signal recorded at 3.8 K after reaching Boltzmann equilibrium)
- $Background$ NMR Signal received from the empty sample cup (Recorded at 3.8 K with same parameters than the Thermal Equilibrium)

- P Polarization.
- P_{eq} ^{1}H polarization due to Boltzmann equilibrium at a given temperature and magnetic field (0.19 at 3.8 K and 7.05 T)
- I Intensity of the integrated spectra.
- G Receiver Gain (dB).
- θ Pulse angle (Calibrated through a nutation experiment).
- NS Number of scans.

1.16 ^{13}C relaxation measurement

The pulse angle of the solid state ^{13}C pulse is measured by monitoring the signal loss under the effect of a large train of pulses. The procedure consists first in hyperpolarizing the ^{13}C spins by CP-DNP so as to obtain a high SNR. Then the signal is acquired by trains of 64 scans, with minimal delay between the acquisitions. Each block of 64 transients is summed and saved in a pseudo 2D experiment. Taking into account the time that the spectrometer needs to store the data, 128 acquisitions are performed in 34 s. For a pulse angle expected in the order of $\sim 5°$, a train of 64 pulses diminished the magnetization from 1 to $(\cos 5°)^{64} \approx 0.78$. After the 128th acquisition, the remaining magnetization is $(\cos 5°)^{64*128} \approx 0$.

It can be shown that the signal intensity along the experiment is given by:

$$S_k/S_0 = \left(\cos\alpha\, e^{-\tau/T_1}\right)^{kN} \approx (\cos\alpha)^{kN} \quad \text{(Supplementary Equation 3)},$$

where S_0 and S_k are the signal intensities of the first and the kth spectra, assuming $k \in [\![0, N-1]\!]$ with N being the number of spectra and where τ, T_1 and α are the time between acquisition blocks, the longitudinal relaxation time constant and the pulse angle. As the longitudinal relaxation time constant T_1 is in the order of hours, the whole loss of magnetization during this procedure is attributed to the effect of the pulses, which allows the simplification of the equation above. This simple equation is fitted to the decay induced by the effect of the pulses with α as free parameter, leading to a precise measurement of the pulse angle.

This allows to compare signal integrals between spectra that were recorded with different number of scans. It can be shown that the signal intensity of two spectra recorded with different number of scans (assuming that each scan destroys a portion $1 - \cos\alpha$ of the magnetization and that no other mechanisms affect the magnetization) is given by:

$$\frac{S_1}{S_2} = \frac{\cos\alpha^{N_1} - 1}{\cos\alpha^{N_2} - 1} \quad \text{(Supplementary Equation 4)},$$

where N_1 and N_2 are the number of scans leading to signal intensities S_1 and S_2, respectively. For example, in the case of a pulse angle of 4.3°, the ratio between signals acquired 64 and 1 scans is not 64 but:

$$\frac{S_1}{S_2} = \frac{\cos^{64} 4.3° - 1}{\cos^{1} 4.3° - 1} \approx 58.6 \quad \text{(Supplementary Equation 5)}$$

The relaxation T1 of the carbon measured at 3.8 K and 7.02 T has been determined using a Matlab fitting script and the following formula:

$$P(t) = P_0 \times e^{\left(\frac{-t}{T_1}\right)} \times (\cos\alpha)^{\left(\frac{NS \times t}{D1}\right)} \qquad \text{(Supplementary Equation 6)}$$

with:

- t Time (hours).
- $P(t)$ Polarization at time t. and at the beginning of the experiment.
- P_0 Polarization at time t=0.
- T_1 ^{13}C Relaxation typical time (hours).
- α Pulse angle (4°).
- NS Number of scans.
- $D1$ Delay between acquisitions (30 minutes).

1.17 Calculation of enhancements after dissolution

Enhancements/polarizations values were calculated in three steps, and first by calculating analyte concentrations in the tube, using the following formula:

$$C_H = C_{Ref} \times \frac{I_H \times NS_{Ref} \times RG_{Ref}}{I_{Ref} \times NS_H \times RG_H} \qquad \text{(Supplementary Equation 7)}$$

with:

- H Proton experiment performed on dissolved solution after let the solution reach the Boltzman equilibrium. In this experiment we observed the formate signal.
- Ref Proton experiment performed on a reference containing 1 M of formate in a fully deuterated solvent.

- C Concentration of formate in moles.
- I Absolute values of formate signal integrals.
- NS Number of scans.
- RG Receiver gain (dB) of each experiments.

Concentration of formate in tube after dissolution have been calculated to be 5 mM and due to the huge signal of water overlapping the signal of the acetate, we assumed to consider concentrations of formate and acetate as equal.

^{13}C enhancements were then calculated the following way:

$$\varepsilon = \frac{I_{Hyp} \times C_{Ref} \times NS_{Ref} \times RG_{Ref}}{I_{ref} \times C_{Hyp} \times NS_H \times RG_H} \qquad \text{(Supplementary Equation 8)}$$

with:

- Hyp ^{13}C signal obtained just after dissolution on formate and acetate.
- Ref ^{13}C reference signal obtained on a reference containing 1 M of formate in a fully deuterated solvent.

- C Concentrations in moles.
- I Absolute values of formate/acetate signal integrals.
- NS Number of scans.
- RG Receiver gain (dB) of each experiments.

1.18 HYPOP-I composition

**Final radical concentration mmol g^{-1}**	**Mass weighted**					**Initial concentration of amino TEMPO (µmol g^{-1})**	**Radical survival yield**
	PPG (g)	DGEBA (g)	amino TEMPO (mg)	IPDA (g)	Total (g)		
**16**	5.1	0.712	7.8	0.1805	6	50.6	33.6 %
**26**	5.1	0.713	11.6	0.182	6.01	76.6	37.9 %
**45**	5.1	0.737	18.2	0.173	6.03	114.5	38.4 %
**62**	5.115	0.7144	26.3	0.171	6.03	168.4	37.4 %
**93**	5.1	0.696	38.7	0.166	6.00	250.9	37.9 %
**116**	5.12	0.689	57.9	0.155	6.02	374.9	30.9 %
**192**	5.1	0.657	87.4	0.141	6.00	564.9	33.8 %
**285**	5.117	0.654	130	0.122	6.02	837.8	34.1 %

***Supplementary Table 2**: Composition of HYPOP-I samples. The composition selected for HYPOP-A sample is highlighted.*

2 Supplementary Discussion

2.1 SEM analyses of porous samples with varying compositions

Solvent: Polypropylene Glycol 1000 g/mol at 50%$_{wt}$

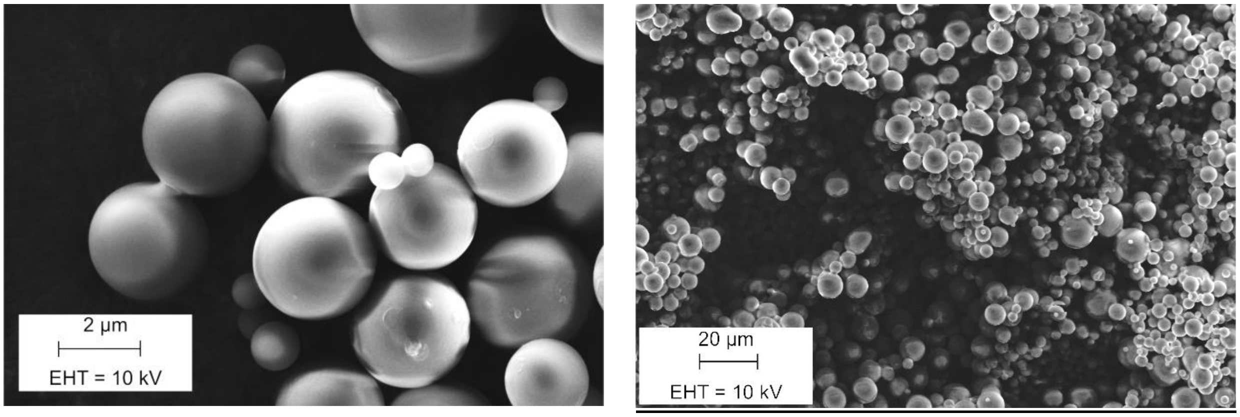

Solvent: Polypropylene Glycol 725 g/mol at 60%$_{wt}$

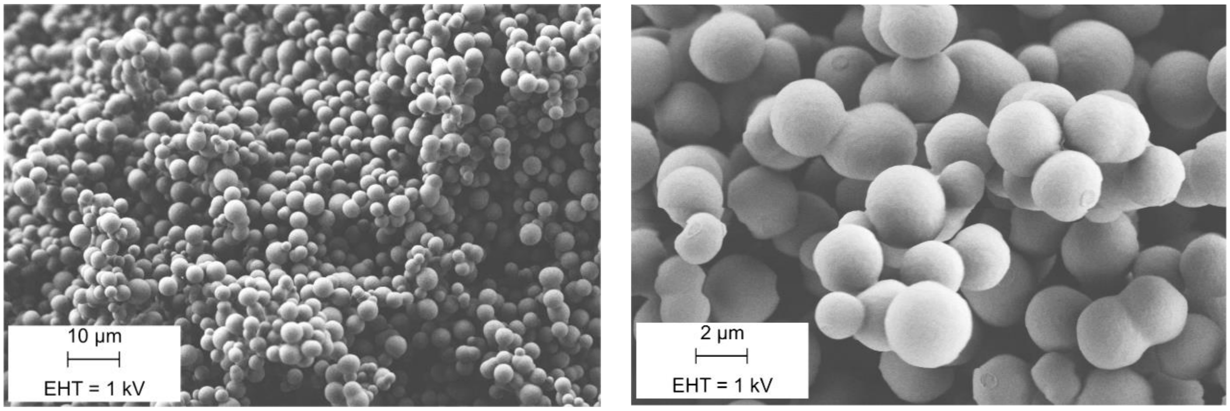

Solvent: Polypropylene Glycol 400 g/mol at 85%wt

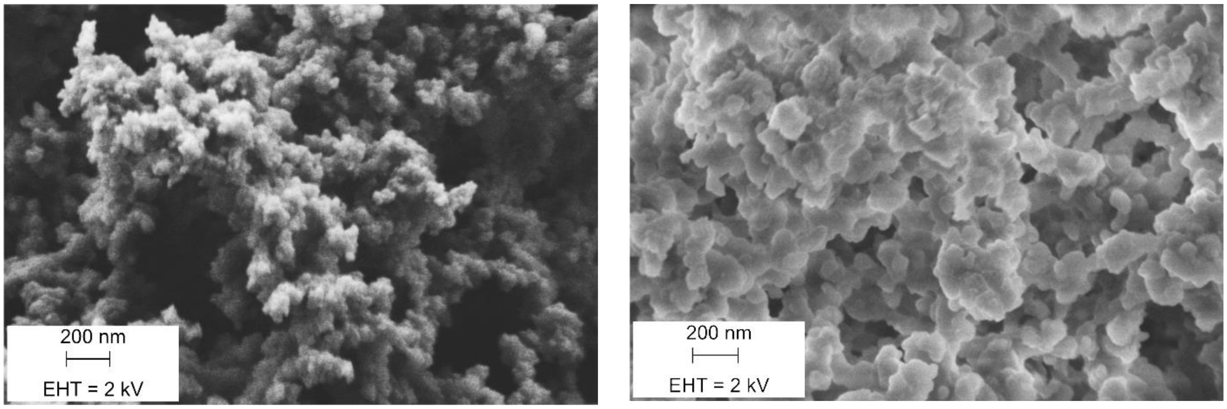

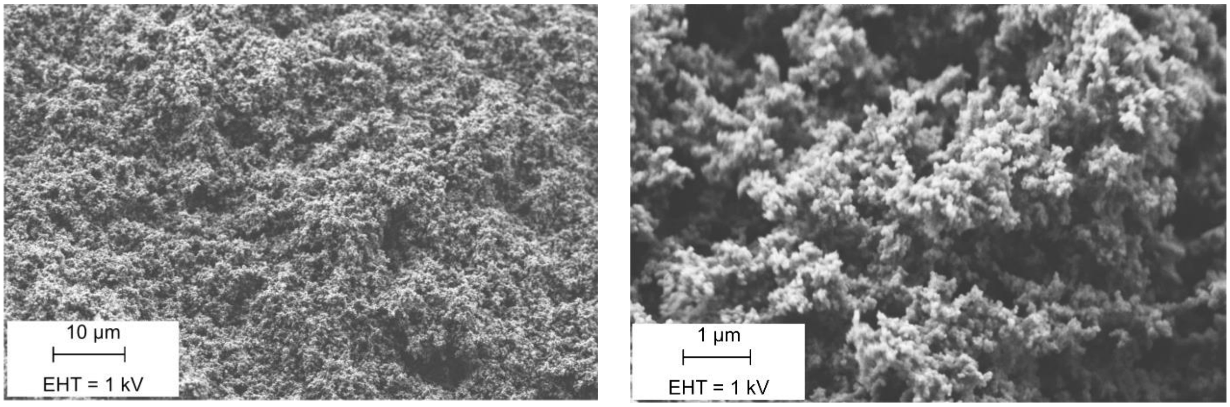

Solvent: Polypropylene Glycol 200 g/mol at 90%wt

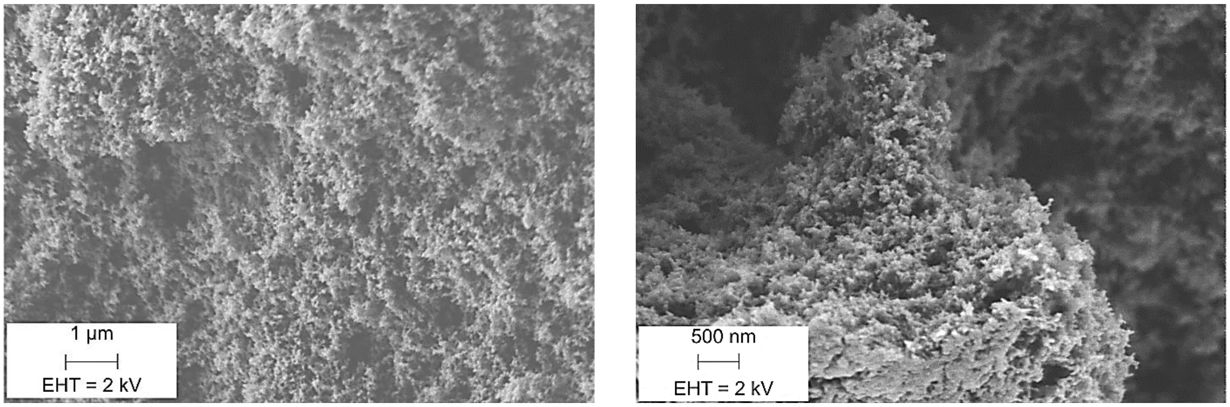

2.2 SEM analyses of HYPOP-I samples

Polymer powder [250µm-500µm] particle size

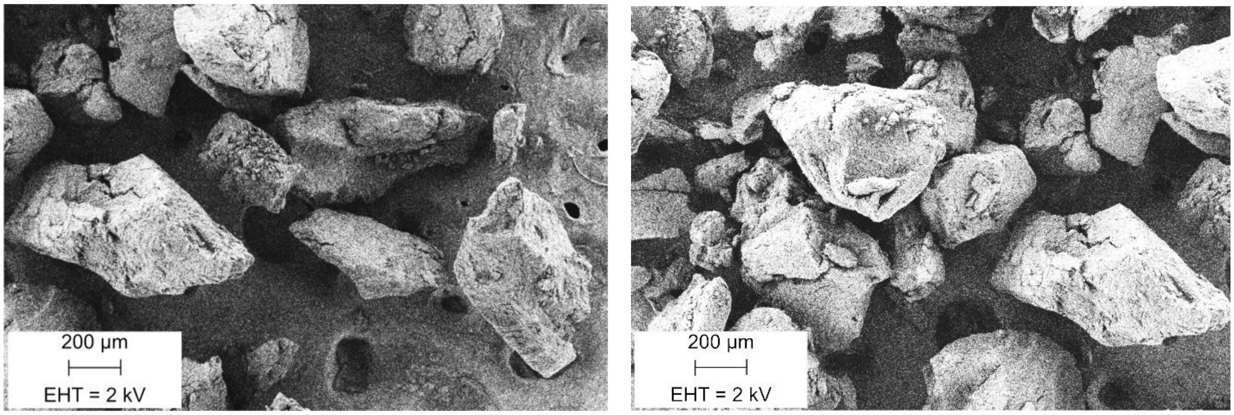

285 µmol g^{-1}

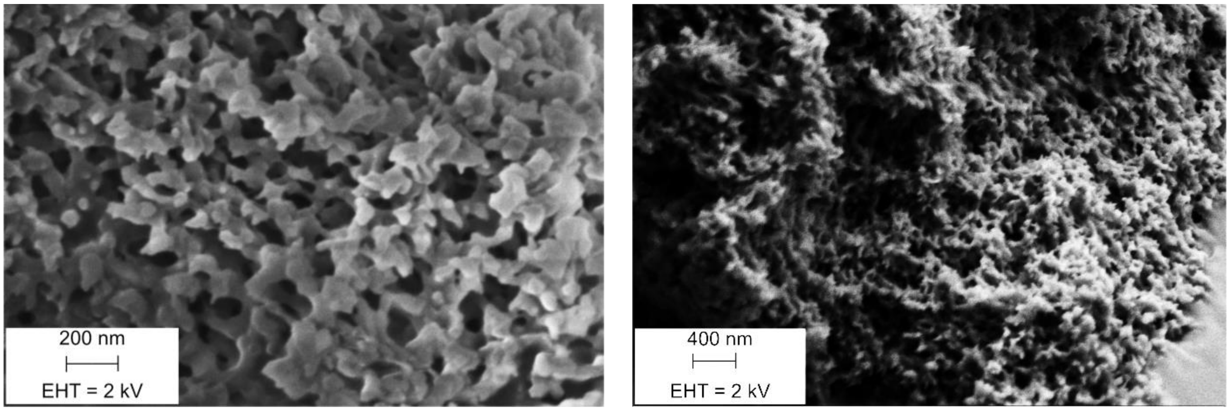

192 µmol g^{-1}:

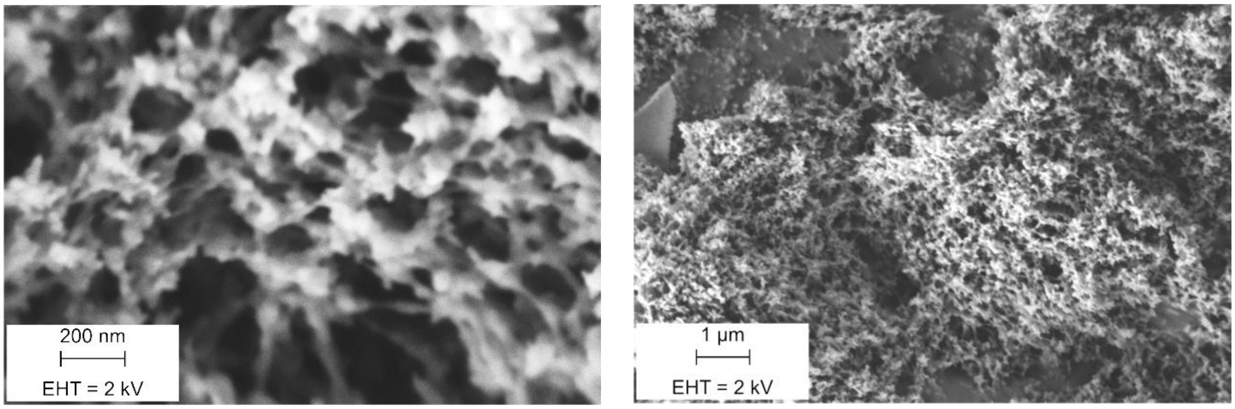

93 μmol g^{-1}

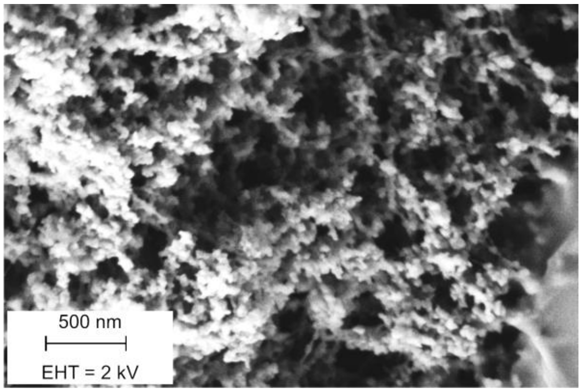

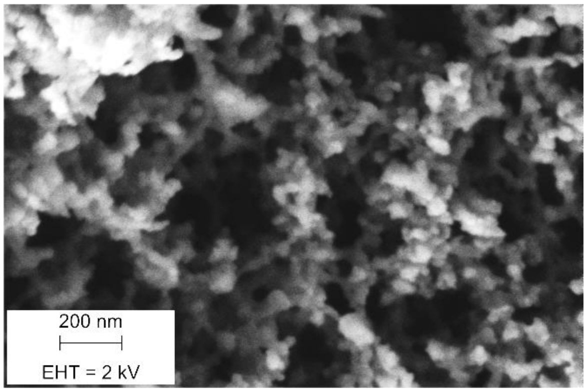

63/ 62 μmol g^{-1}

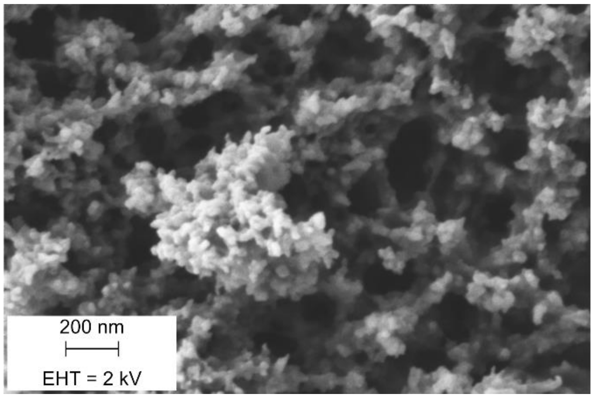

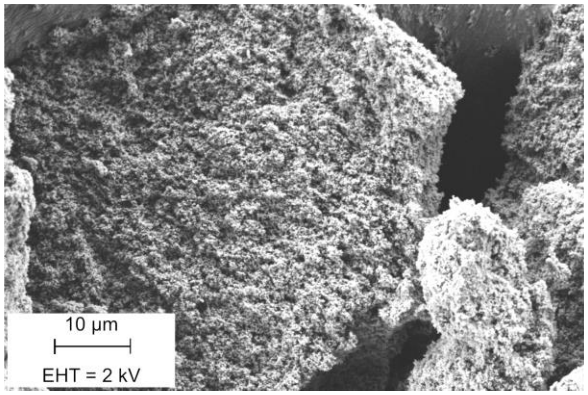

45 μmol g^{-1}

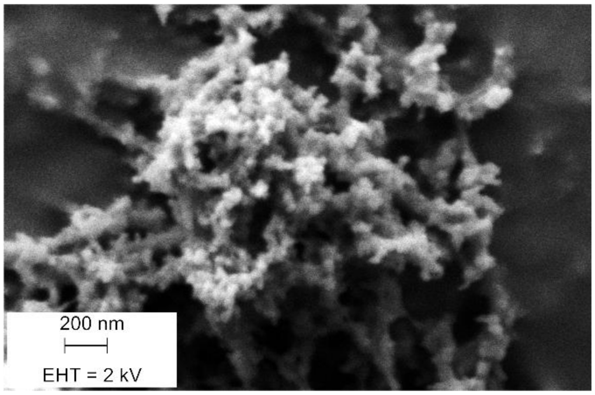

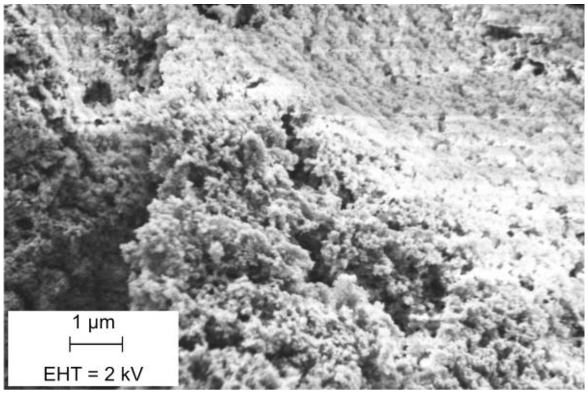

26 µmol g^{-1}:

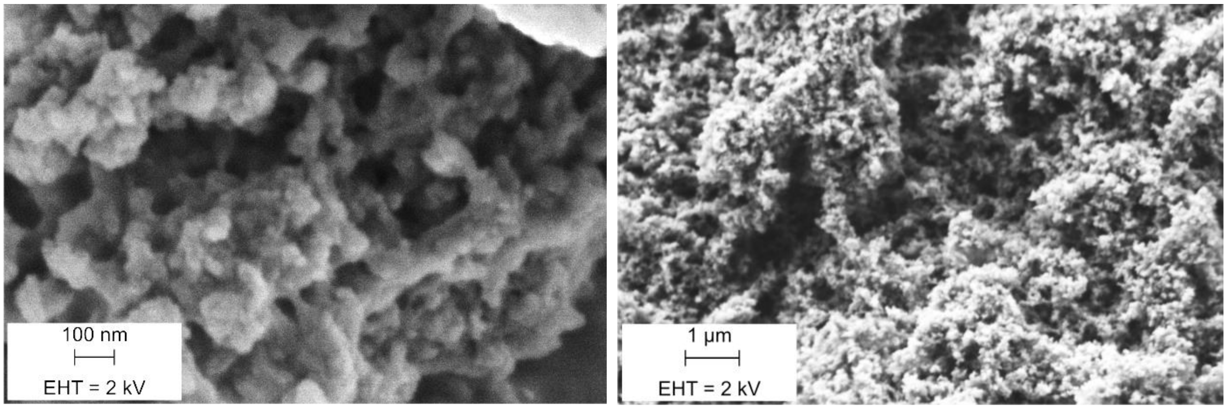

16 µmol g^{-1}:

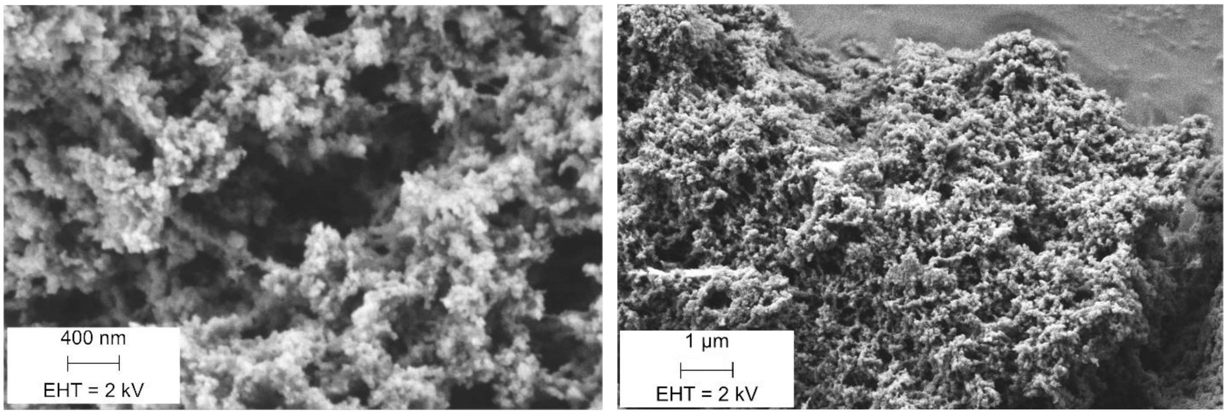

Supplementary Figure 2: *SEM Images of HYPOP-I samples used in DNP / EPR.*

2.3 Monitoring of the curing by rheology

We monitored the curing kinetics of a TEMPO-free sample comprising 85%$_w$ of PPG 400 g/mol as described above (*Supplementary Figure*). While the initial phase separation, indicated by a first increase of the viscosity, appears after 3 h of reaction, the subsequent increase of the storage modulus (G'), indicative of the formation of a network between aggregated particles, occurs about 30 min later and slowly continues to progress, reaching a value at 90% of maximum after 15 h.

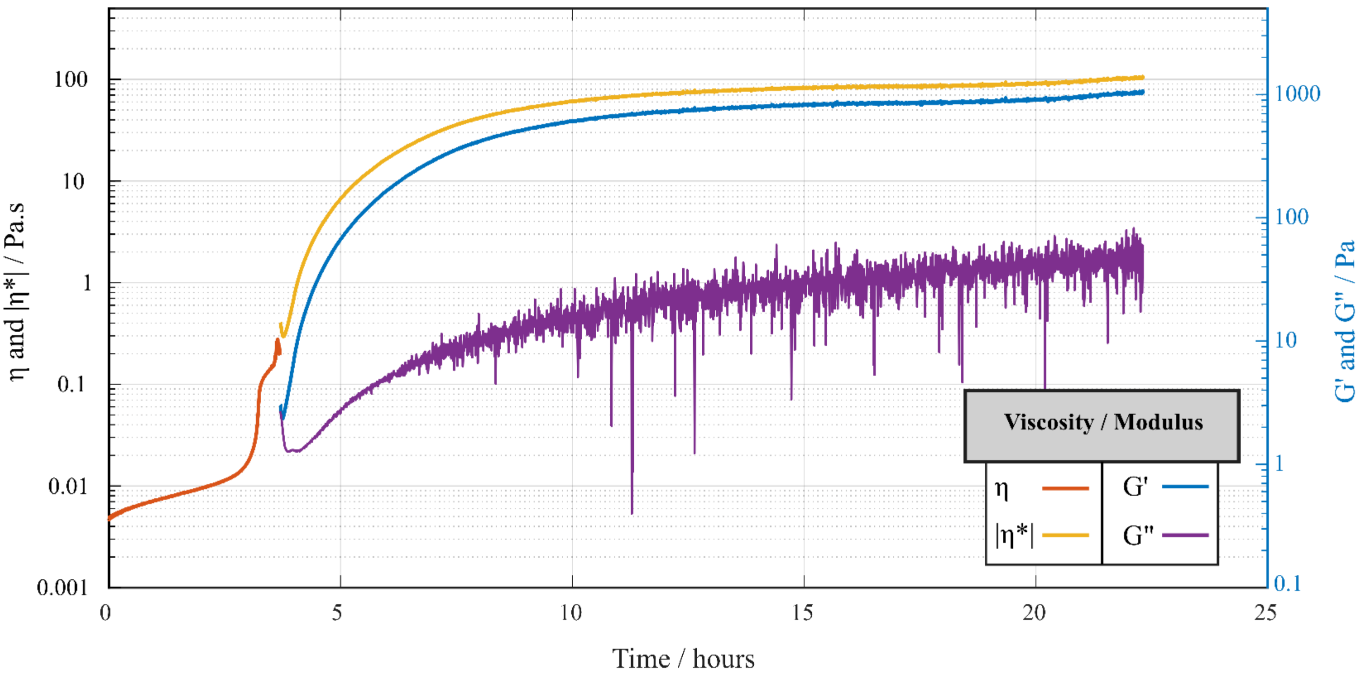

***Supplementary Figure 3**: In situ monitoring of the curing process using 85%$_w$ of PPG 400 g/mol. Up to 3h (red curve), constant shear rate of 10 s^{-1} is applied. In proximity to the gel point, oscillatory shear (1 Pa, 10 rad.s^{-1}) is applied.*

2.4 Mercury Intrusion Porosimetry

In contrast to SEM observation that could not demonstrate significant changes of morphologies within the HYPOP-I series, mercury intrusion porosimetry indicates a significant shift in the size distribution of pores when large amounts of amino TEMPO are used (*Supplementary Figure* 4).

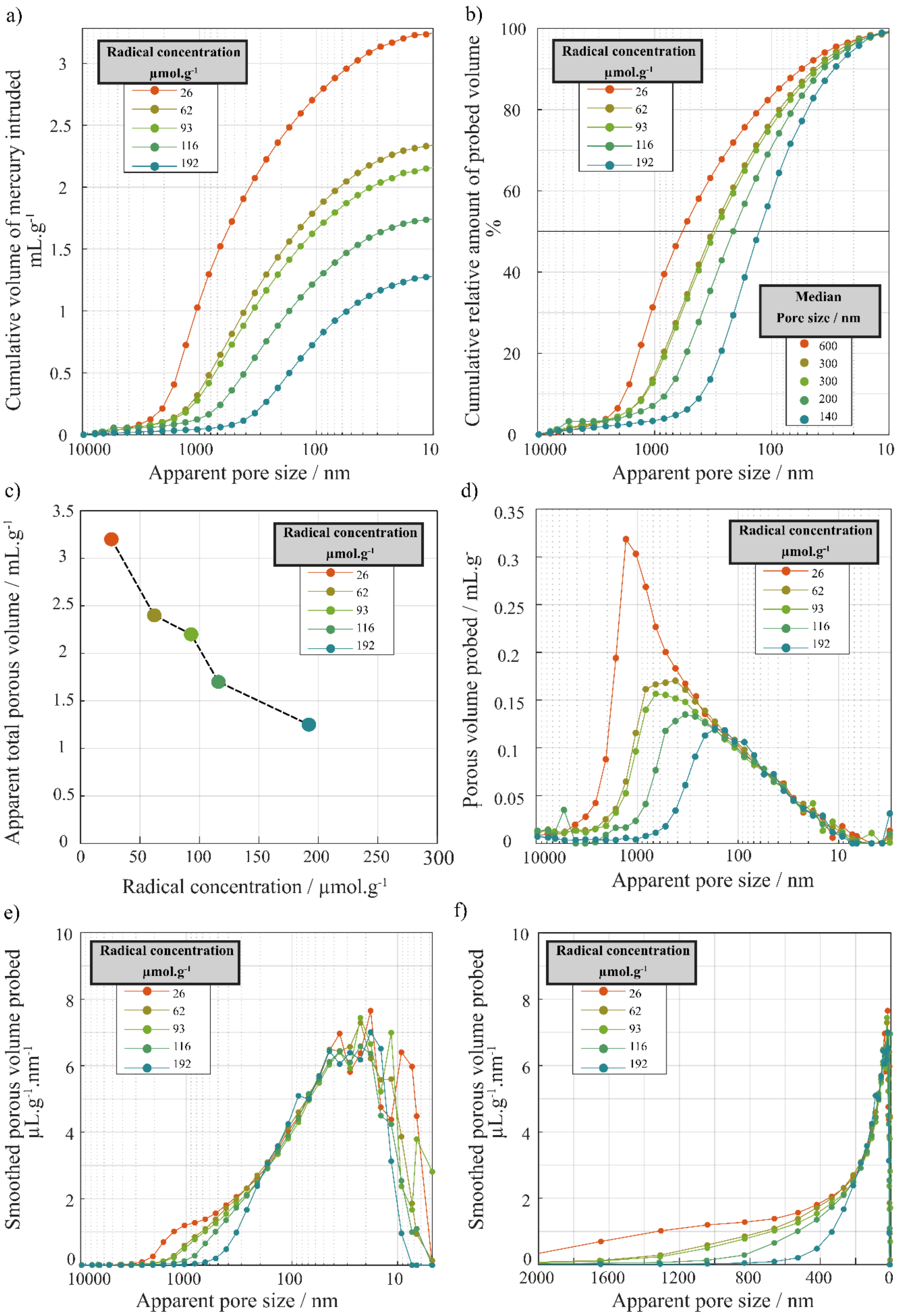

***Supplementary Figure 4**: (a) Cumulative volume of mercury introduced in polymers. (b) Relative volume filled by mercury in function of apparent pore size. (c) Total volume probed in function of radical concentration. (d) Distribution of the probed volume depending on apparent pore size. (e) Smoothed distribution of the probed volume (f) Linear scale of (e).*

2.5 Nitrogen physisorption

Mercury intrusion porosimetry may induce isostatic compression of the porous samples, especially when pore sizes lower than 100 nm are probed (this corresponds to Hg pressures above 10 MPa). Thus, we also complemented the porosity characterization with nitrogen physisorption (*Supplementary Figure 5*). While this technique is less invasive in terms of pore deformation, it can only probe pore sizes in the 1-100 nm range. The type-II isotherms displayed by all HYPOP-I samples confirms that these samples are essentially macroporous (pore size above 50 nm) and indicate significantly lower pore volume for samples containing 192 and 285 µmol/g of radicals.

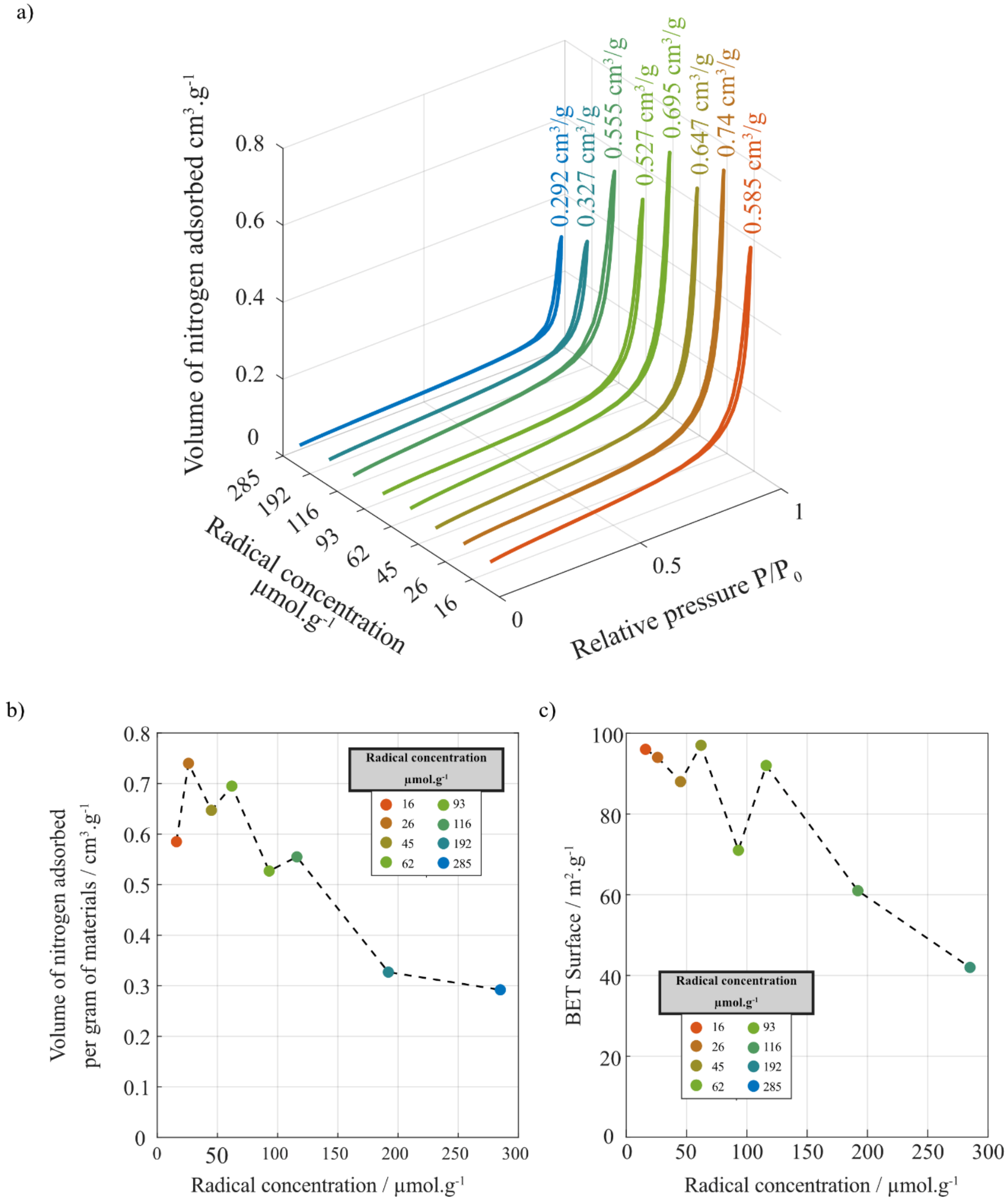

Supplementary Figure 5: *(a) Nitrogen Adsorption/Desorption isotherms for the HYPOP-I samples (labels corresponds to the cumulative volume of nitrogen adsorbed in pores lower than 100 nm). Impact of the radical concentration of HYPOP-I samples on: (b) The global probed pore volume. (c) The BET specific surface area.*

2.6 EPR Calibration

To properly calibrate our measure of radical concentration, we prepared a large range of solid dilutions of fresh TEMPO in KBr and analyzed them at room temperature. Those standards were mixed then ground before being used in EPR. After analysis of results, we obtained the following calibration curve (*Supplementary Figure 6*). Uncertainty was calculated by correcting the standard deviation of residuals by the adapted Student factor (degree of freedom: 17 / Risk: 1% / t-factor = 2.55). [2]
Residuals were plotted to check absence of trends that would discredit the affine model.

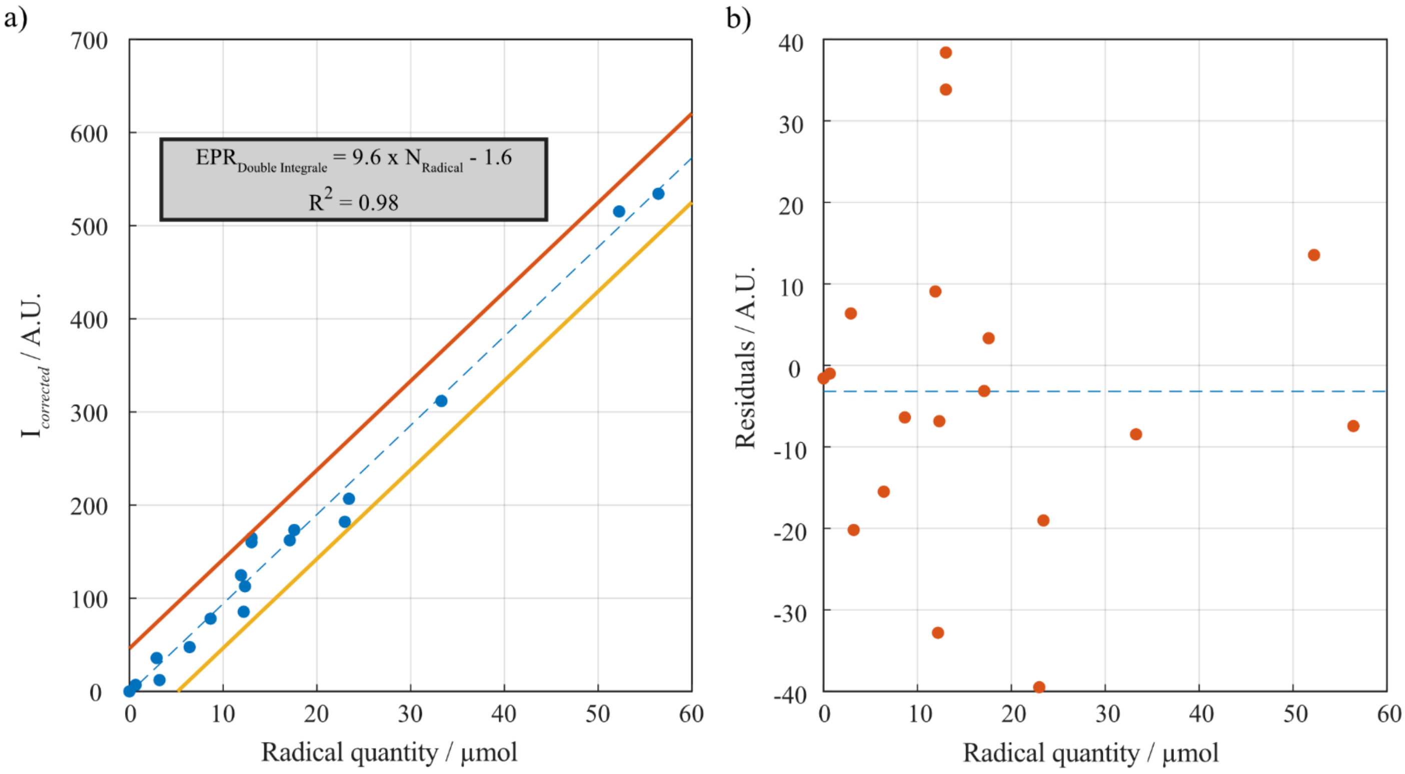

***Supplementary Figure 6**: (a) Calibration curve of radical quantification (b) Residuals.*

2.7 EPR spectra and quantification of radicals

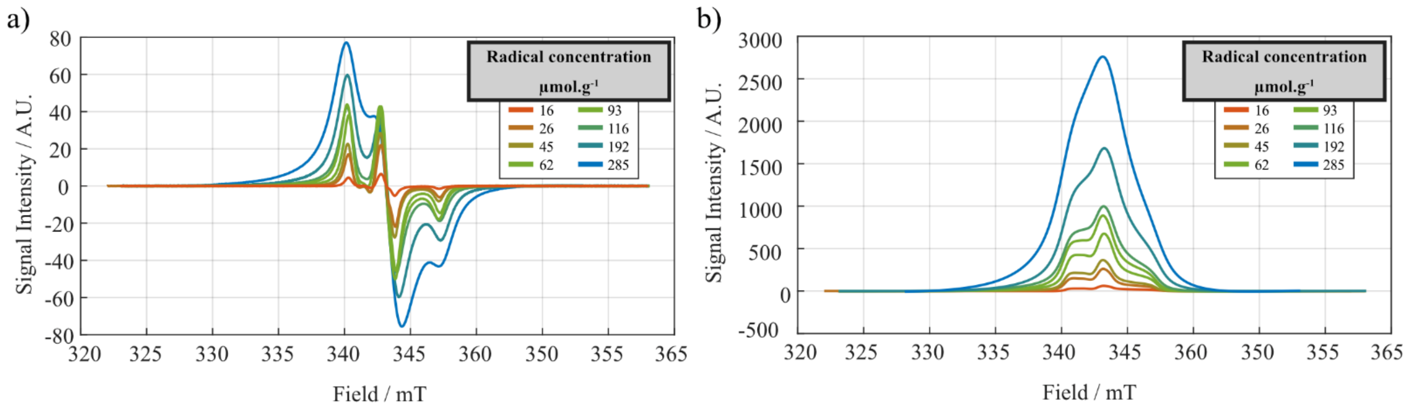

Supplementary Figure 7: *EPR spectra of HYPOP-I samples (a) before and (b) after initial integration and baseline correction.*

Double integration value (AU)	Modulation (G)	Microwave Power (mW)	Q factor	Weight (mg)	I corrected (AU)	Radical Concentraion (µmol g^{-1}), ± 5 µmol g^{-1}
**2900**	1	0.1	5414	21.5	0.7	16
**5800**		0.1	5636	19.2	3.3	26
**14000**		0.1	5945	20.8	7.4	45
**20000**		0.1	5681	21.3	11.1	62
**28000**		0.1	5241	20.8	16.9	93
**50000**		0.1	6020	25.0	26.3	116
**98000**		0.1	6094	28.5	50.9	192
**167000**		0.1	5682	34.7	92.9	285

Supplementary Table 3: *Experimental parameters used for the measure of radical concentration in HYPOP-I samples*

2.8 Thermal equilibrium & DNP buildup

Thermal equilibrium, background and proton DNP build up spectra have been obtained using the following pulse sequence:

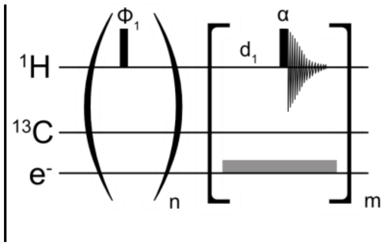

Supplementary Figure 8: *Pulse sequence used to obtain proton DNP build up. Alpha is a small angle pulse (maximum 5°) calibrated through a nutation experiment and n=50.*

2.9 Cross-polarization

Cross-polarization is realized with a homemade coils described in a previous article.[3]

Adiabatic half passage chirp pulse have been built with half WURST pulse, 100 kHz broad with 500 point spread in 175 µs in both channels (150 W for ^{13}C, 12 W for ^{1}H). Contact is realized with 6 ms contact pulses empirically chosen as 100-50-100 pulse on carbon-13 and square pulse on 1H (26 kHz ^{13}C at 150 W and 14 kHz ^{1}H at 7 W).

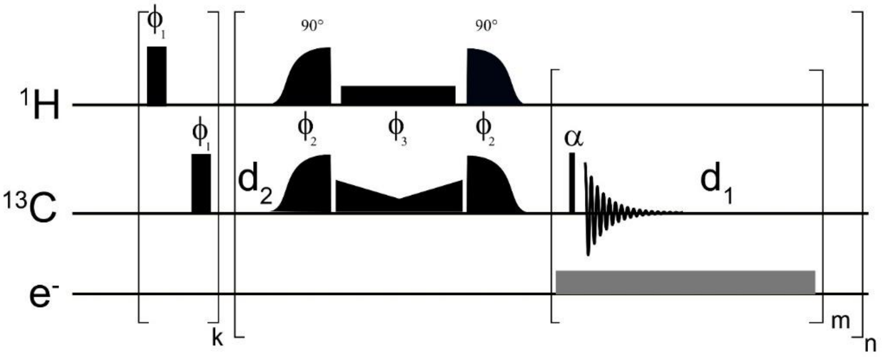

***Supplementary Figure 9**: Pulse sequence for the cross-polarization used in the d-DNP experiments, α=5°, k=50, m=6, n=32, d1=30 s, Φ1=[x,y,-x,-y,-y,y,x,-x,-y,-y,-x], Φ2=[x], Φ3=[y].*

2.10 Swelling measurements

Ideally, the target solutions should only impregnate the porous volume of HYPOPs. However, potential affinity of solvents or analytes for the epoxy may also induce diffusion into the polymer particles and an overall swelling of the porous material. Not only is this swelling detrimental to the extraction yield of hyperpolarized analytes, but it may also drastically reduce the hyperpolarization lifetime by facilitating relaxation towards PAs through ^{13}C-^{13}C spin diffusion or weaken the mechanical properties of the polymer network. To estimate this swelling phenomenon, we measured the mass before and after impregnation of a porous monolith (between 250 and 550 mm^3 dry) immersed in various solvents for two hours. By comparing masses before and after with respect to solvent density it is possible to obtain a global impregnation volume that includes both the porous and swelling volumes. PPG-4000 was used as a reference solvent, able to impregnate the porous volume but unable to swell the epoxy particles.

Impregnated volume was calculated with the formula:

$$V_i = \frac{(m_f - m_i)}{\rho_s \times m_i} \quad \text{(Supplementary Equation 9)}$$

with:
- V_i Impregnated volume per gram for a given solvent (mL g^{-1}).
- m_i & m_f respectively masses before and after impregnation of the polymer block (g).
- ρ_s Volumic mass of the solvent (g.mL^{-1}).

Swelling volume was calculated with the formula:

$$V_s = V_i - V_{i\,ref} \qquad \text{(Supplementary Equation 10)}$$

with:

- V_s Swelling volume per gram for a given solvent (mL g^{-1}).
- V_i & $V_{i\,ref}$ Respectively impregnated volume per gram for a given solvent and for the reference: PPG-4000 (mL g^{-1}).

Apparent porosity was calculated with the formula:

$$P_{\%} = 100 \times \left(\frac{V_i}{\frac{m_i}{\rho_p} + V_i} \right) \qquad \text{(Supplementary Equation 11)}$$

with:

- $P_{\%}$ Apparent porosity of the polymer (%).
- V_i Impregnated volume per gram for a given solvent (mL g^{-1}).
- m_i Mass before impregnation of the polymer block (g).
- ρ_ρ Volumic mass of the polymer which has been found to be 1.006 (g mL^{-1}).

Percentage of swelling was calculated with the formula:

$$S_{\%} = 100 \times \left(\frac{V_s}{V_i} \right) \qquad \text{(Supplementary Equation 12)}$$

with:

- $S_{\%}$ Part of swelling during impregnation (%).
- V_s Swelling volume per gram for a given solvent (mL g^{-1}).
- V_i Respectively impregnated volume per gram for a given solvent and as reference: PPG-4000 (mL g^{-1}).

Solvent	Initial mass of polymer (mg)	Final mass after impregnation (mg)	Volume impregnated per polymer mass (mL g^{-1})	Swelling (mL g^{-1})	Real Porosity	Apparent porosity	Percentage of swelling
**PPG-4000 (reference)**	110	563	4.1	0	80.4%		0%
**Ethanol**	64.4	342	5.5	1.4		84.5%	25%
**Dimethyl sulfoxide**	96	876	7.4	3.3		88.1%	44%
**Ethanol:water 10:90v**	**75.5**	**394**	**5.1**	**1.0**		**83.6%**	**19%**
**Acetonitrile**	83.5	388	4.6	0.5		82.3%	12%
**Dichloromethane**	112.7	1020	6.1	2.0		85.8%	32%
**Acetone**	82.5	408	5.0	0.9		83.4%	18%

Supplementary Table 4: *Calculation of effective porosity and swelling volumes for the porous sample containing 85%$_{wt}$ of PPG-400.*

2.11 Microwaves optimization at 1.2K and 7.05 T, on HYPOP-I:

Microwaves were optimized on one dry HYPOP, then compare with another impregnated HYPOP without observing any change on optimal frequency. We assumed similar pattern for all polymers (dry and impregnated) and so kept 197630 MHz as microwave frequency for all DNP Build up.

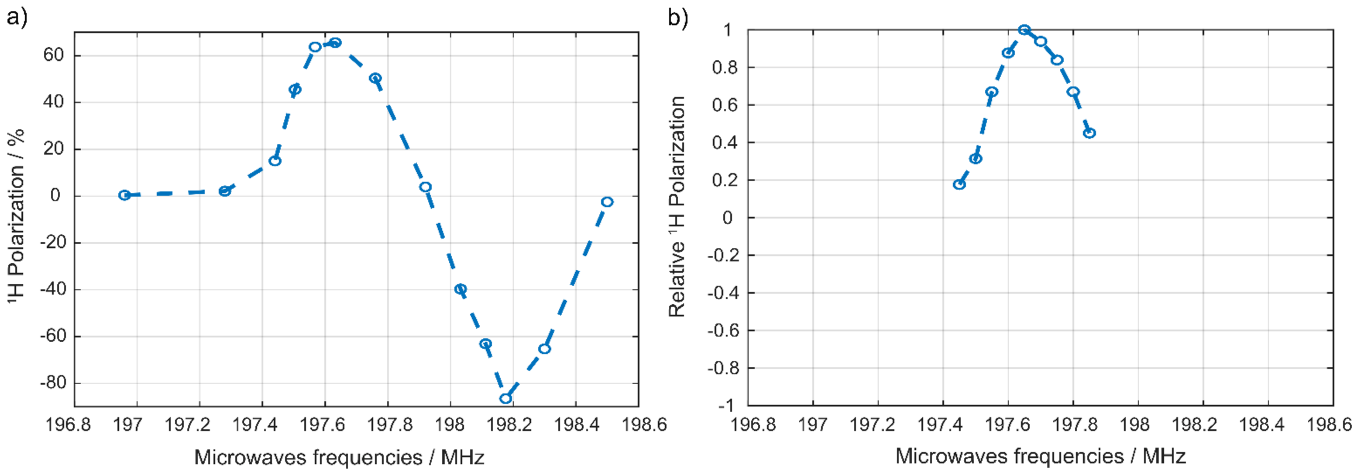

Supplementary Figure 10: *DNP spectra for both dry and impregnated HYPOP. (a) 192 mM HYPOP impregnated with 10 M ^{1}H solution. (b) 285 mM HYPOP dry.*

2.12 Dry HYPOP ^{1}H DNP build-ups

Maximal polarization given below describes only maximal values reached by effective data. In some cases, build up are incomplete, so asymptotic polarization values have been extrapolated from the fits.

* Due to some technical difficulties (wrong microwave frequency was initially used) the ^{1}H build-up of dry HYPOP at 192 µmol g^{-1} (Fig 3a in main text) was performed in two steps. Therefore, final polarization level is accurate while RDNP measurement is not.

Δ Dry HYPOP at 285 µmol g^{-1} gave a thermal signal too weak to provide a reliable measure of the polarization. Thus only R_{DNP} was indicated (Fig 3b in main text).

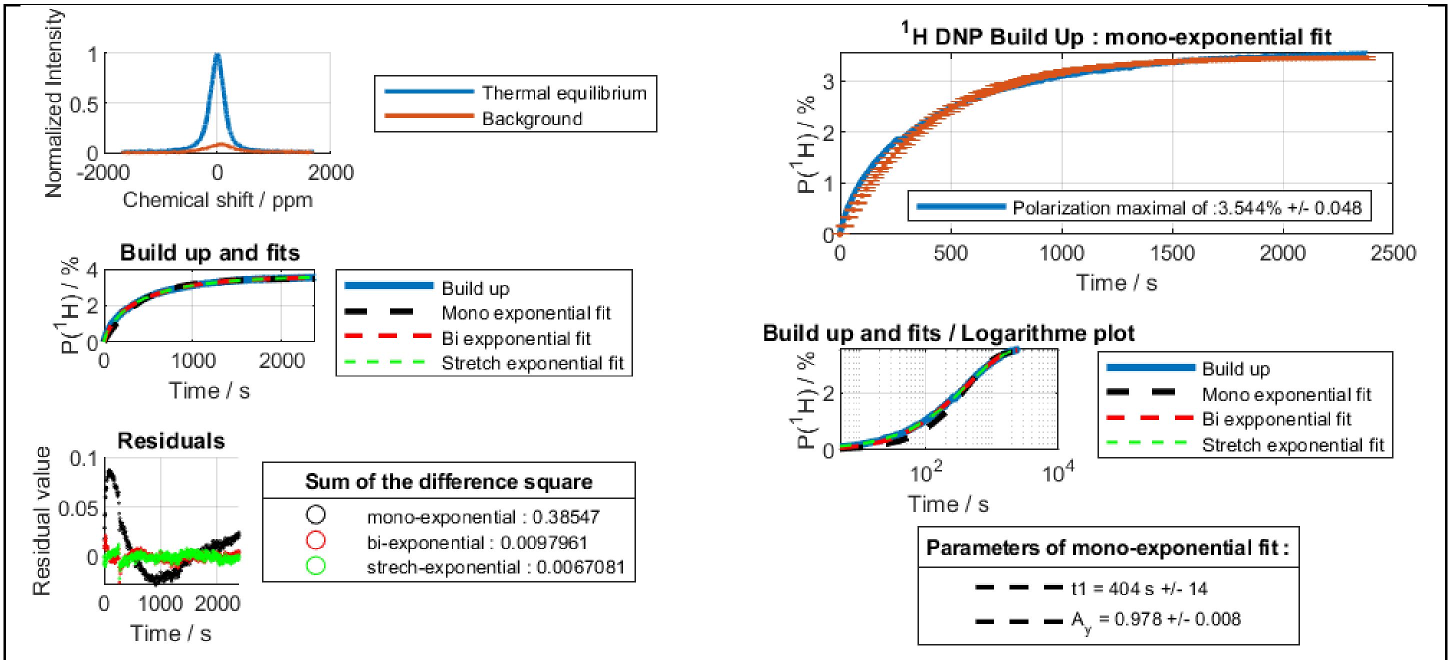

TED 60 / 16 µmol. g^{-1}:

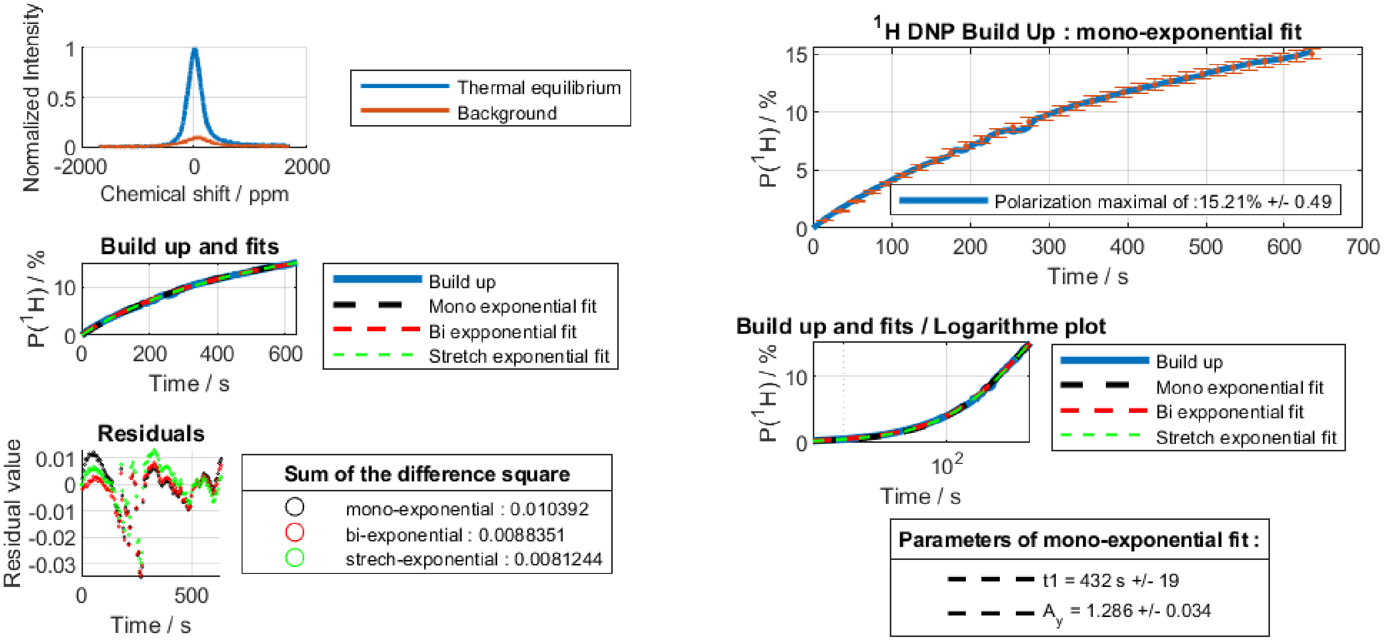

TED 61 / 26 µmol. g^{-1}:

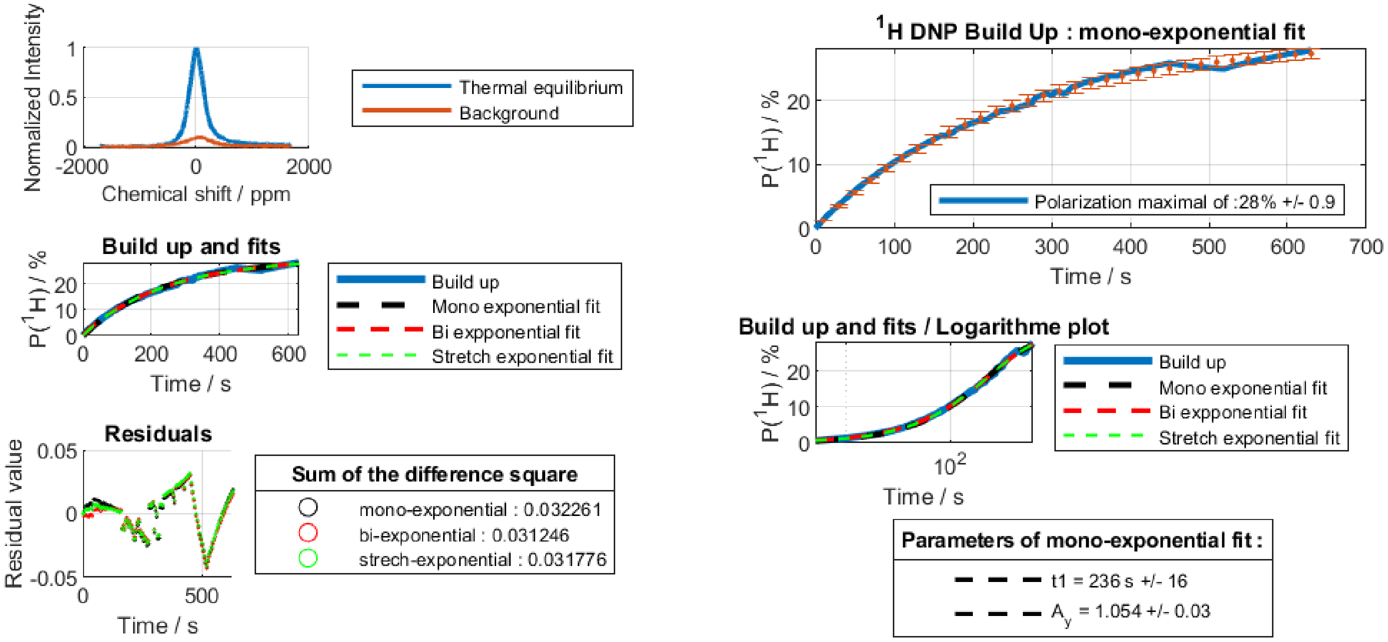

TED 62 / 45 µmol. g^{-1}:

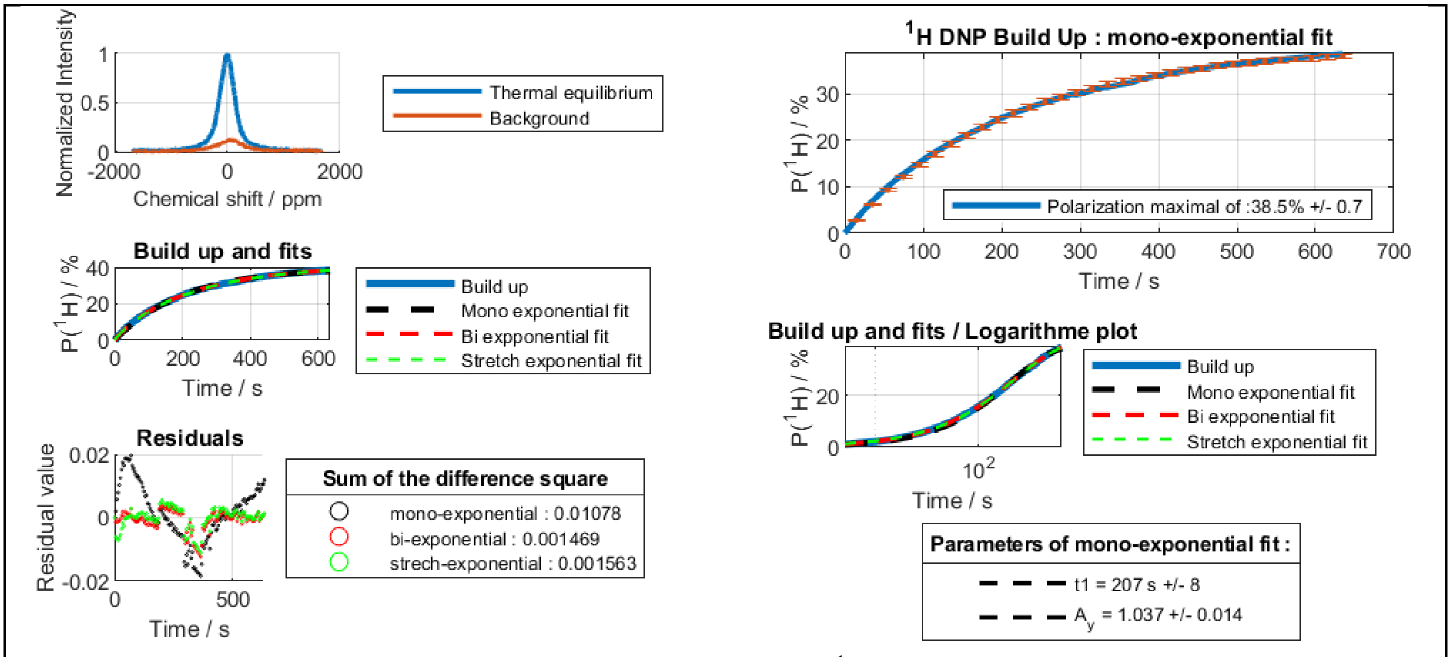

TED 63 / 62 μmol. g^{-1}

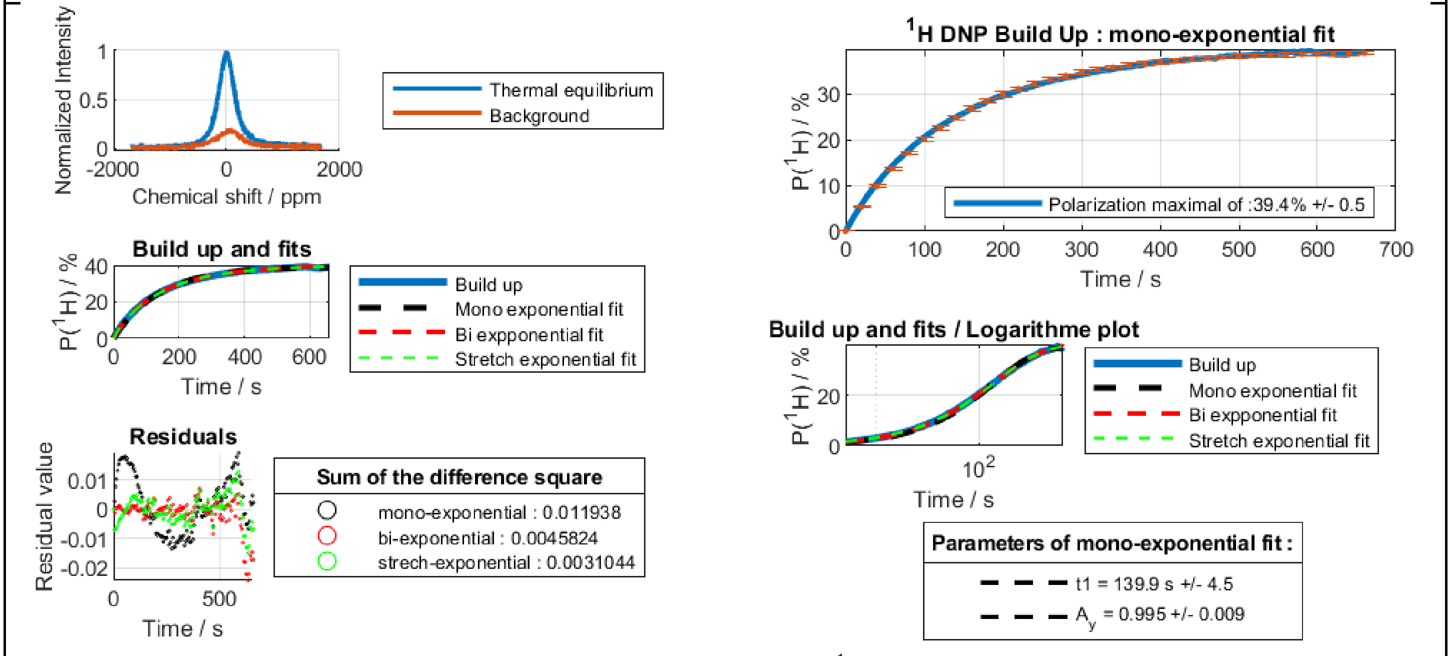

TED 64 / 93 μmol. g^{-1}

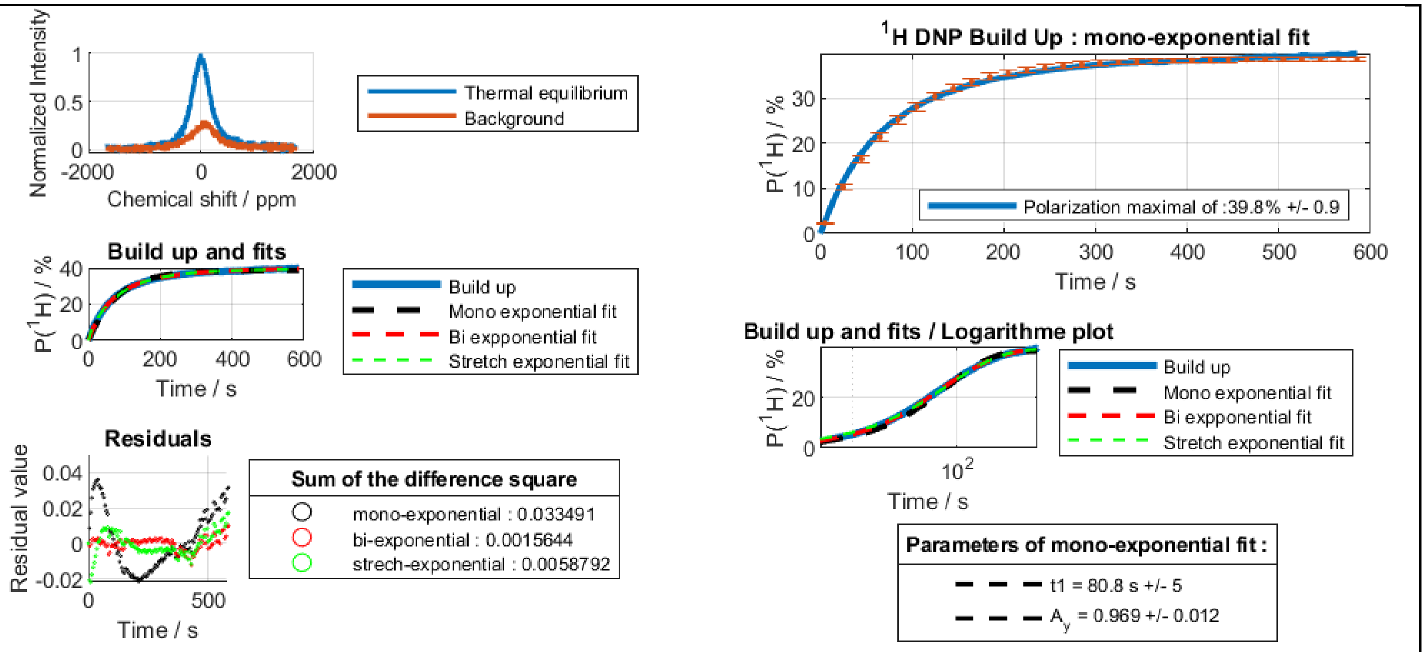

TED 65 / 116 µmol. g^{-1}

Global

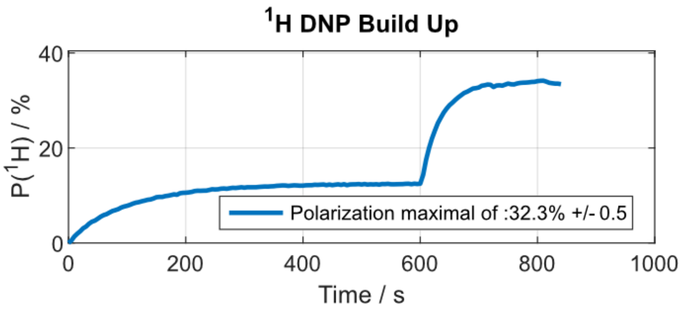

Part 1

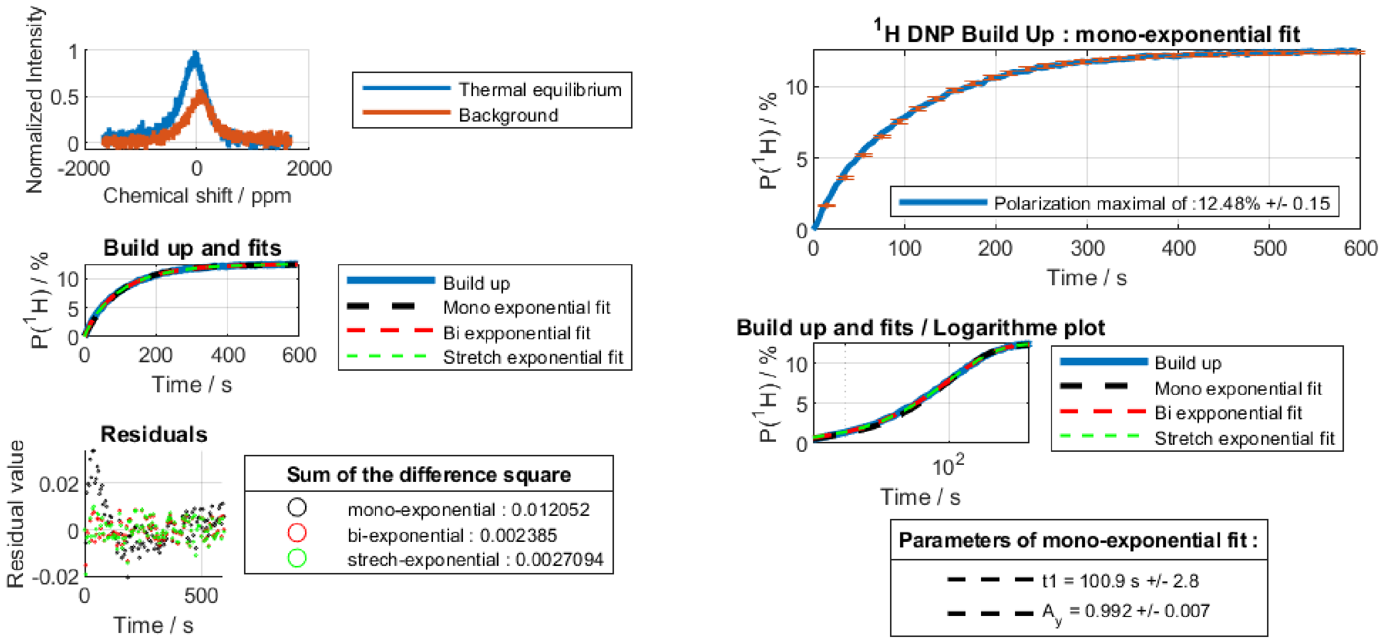

Part 2

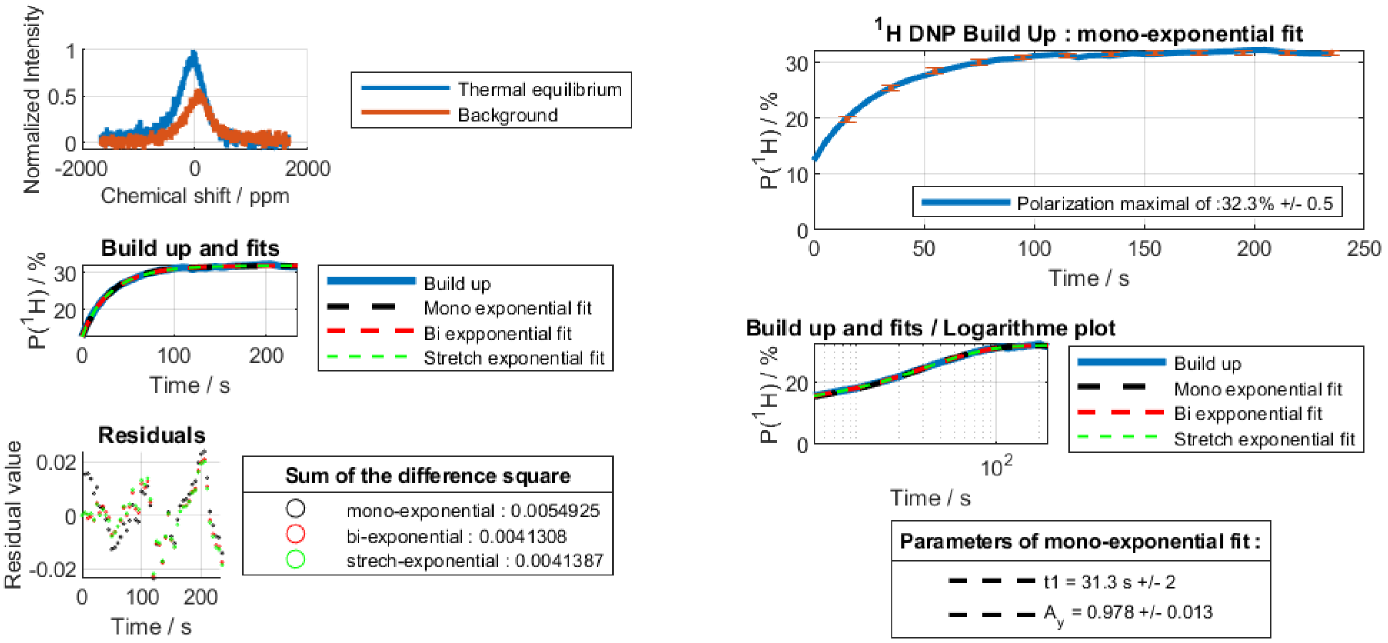

TED 66 / 192 µmol. g^{-1}: Data have been acquired in two time, the first one with the wrong microwave frequency and modulation, the second one with conventional parameters.

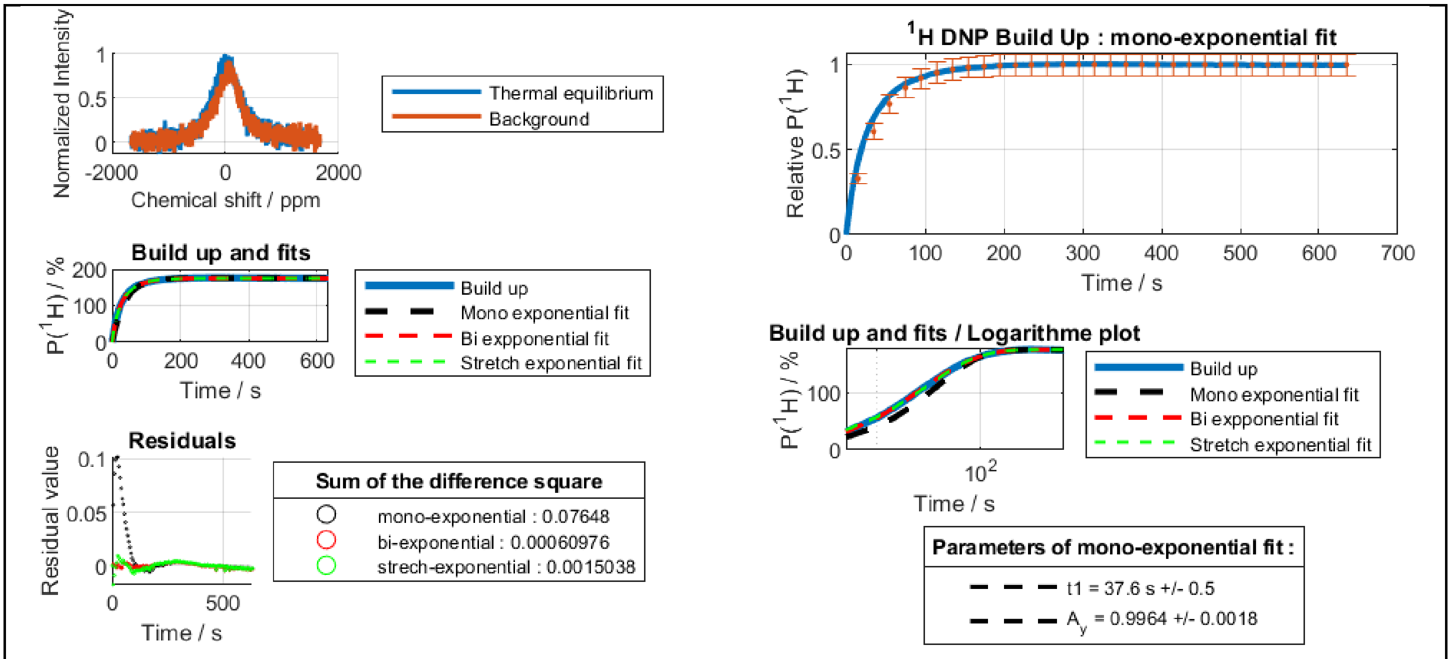

TED 67 / 285 µmol. g^{-1}:

2.13 Impregnated ($10\%_v$ H_2O / $10\%_v$ $ETOD_6$ / $80\%_v$ H_2O) HYPOP ^1H DNP build-ups

Maximal polarization given below describes only maximal values reached by effective data. In some cases, build up are incomplete, so asymptotic polarization values have been extrapolated from the fits.

▪ Due to a technical mistake, impregnated HYPOP at 285 µmol g^{-1} has been polarized without the same microwaves modulation and so its RDNP is not consistent with others (Fig 3a in main text).

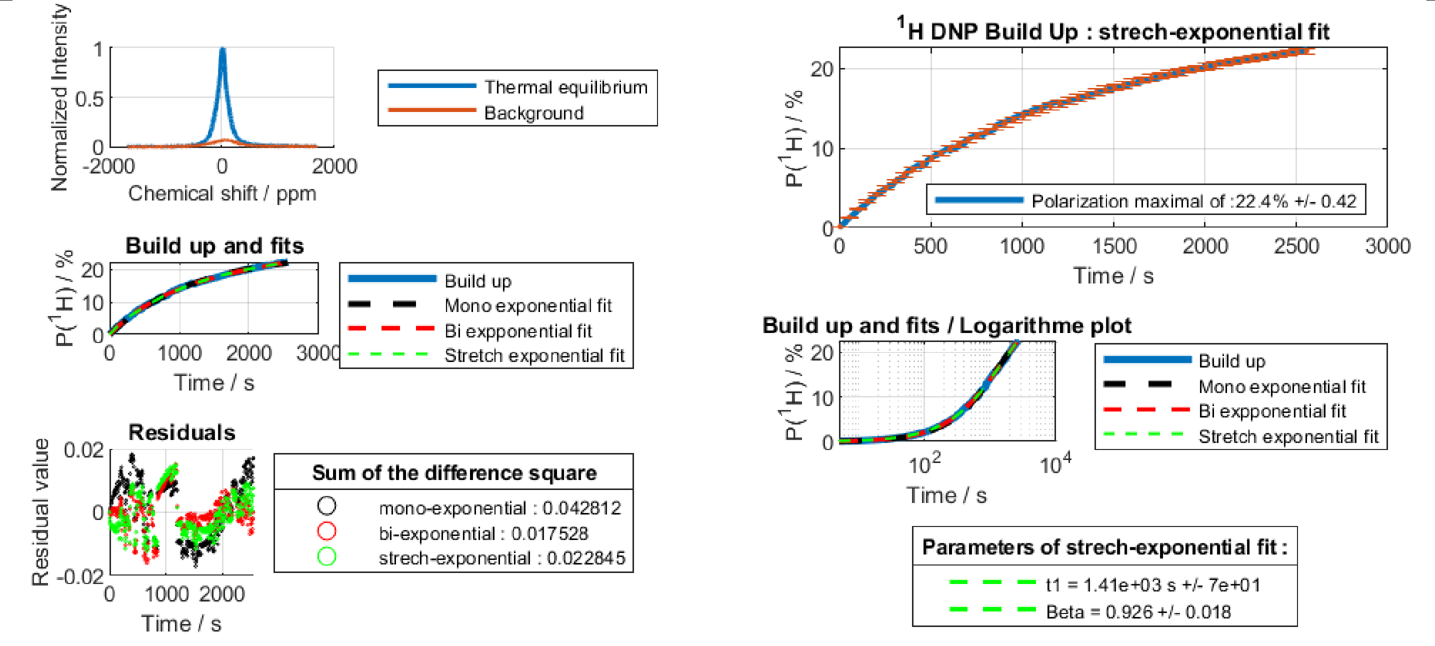

TED 60 / 16 µmol. g^{-1}:

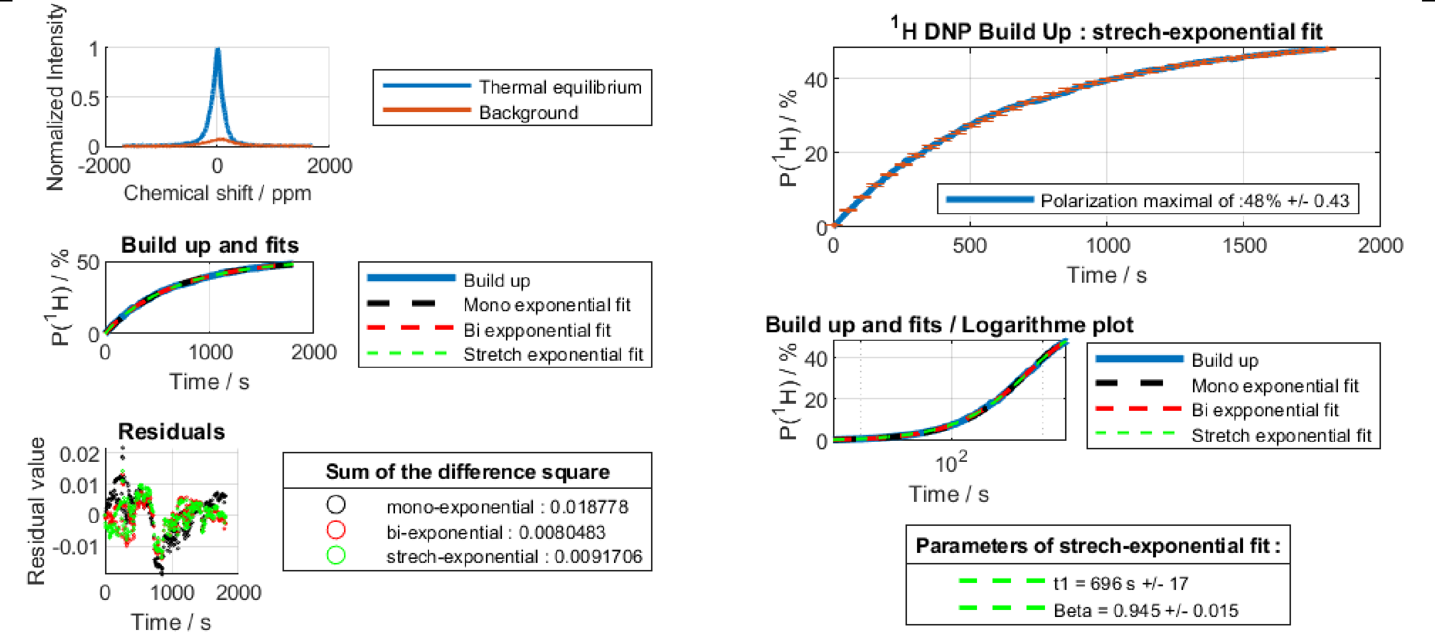

TED 61 / 26 µmol. g^{-1}:

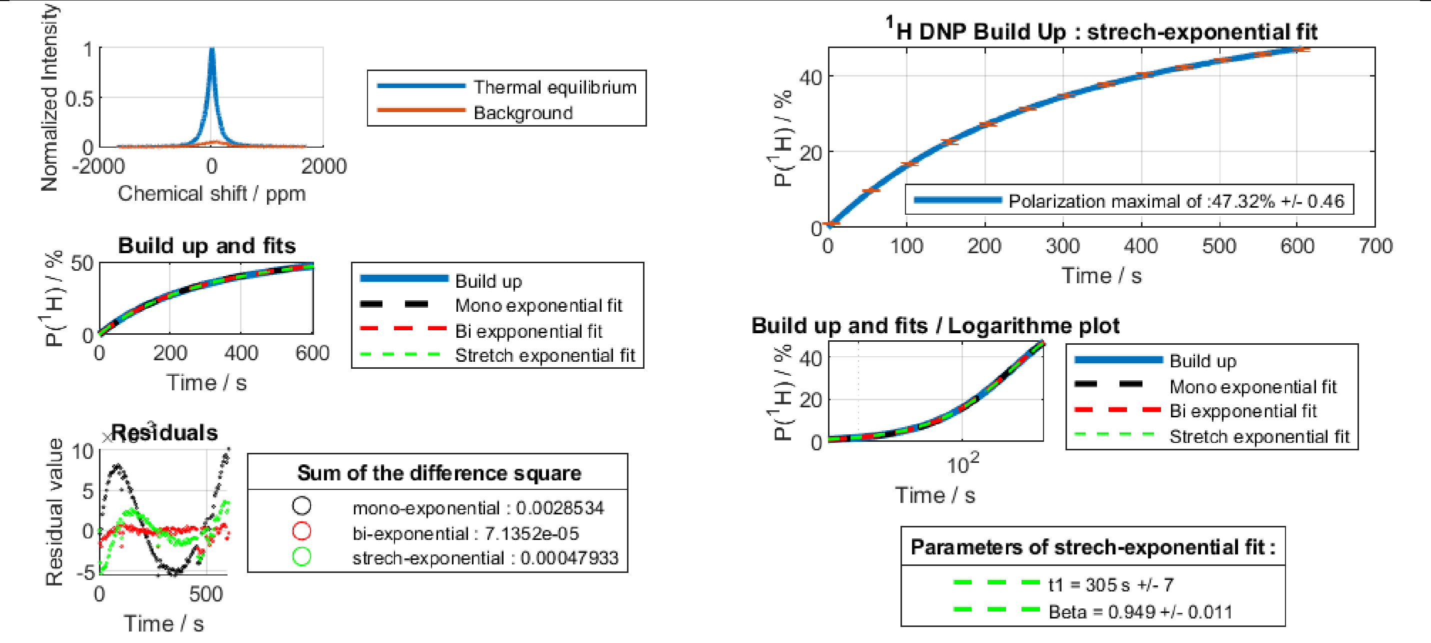

TED 62 / 45 µmol. g-1

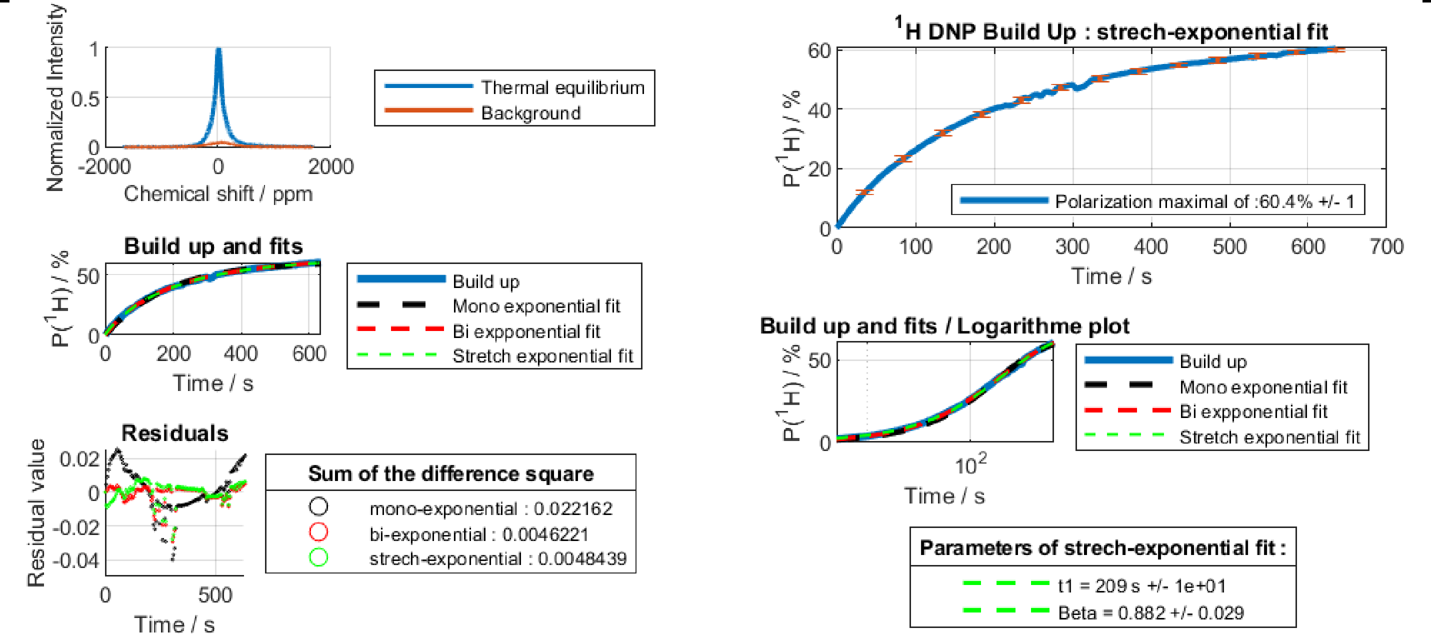

TED 63 / 62 µmol. g-1:

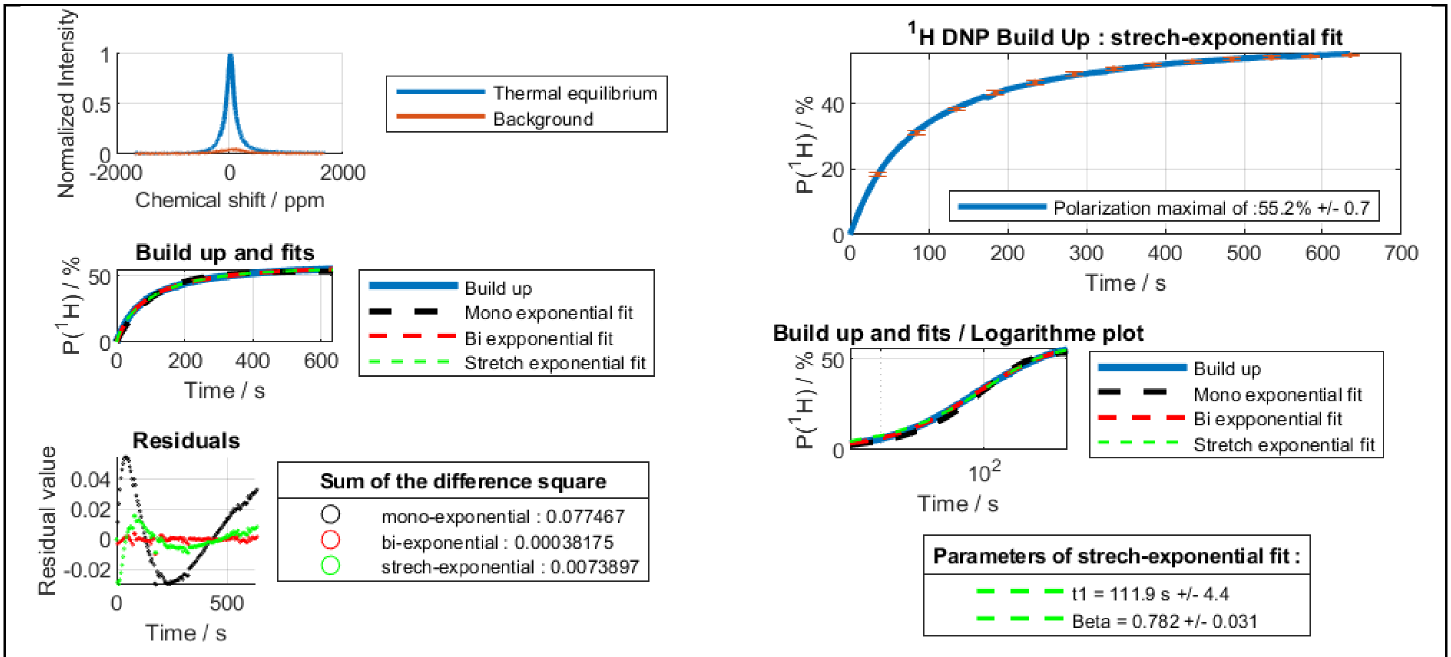

TED 64 / 93 µmol. g-1

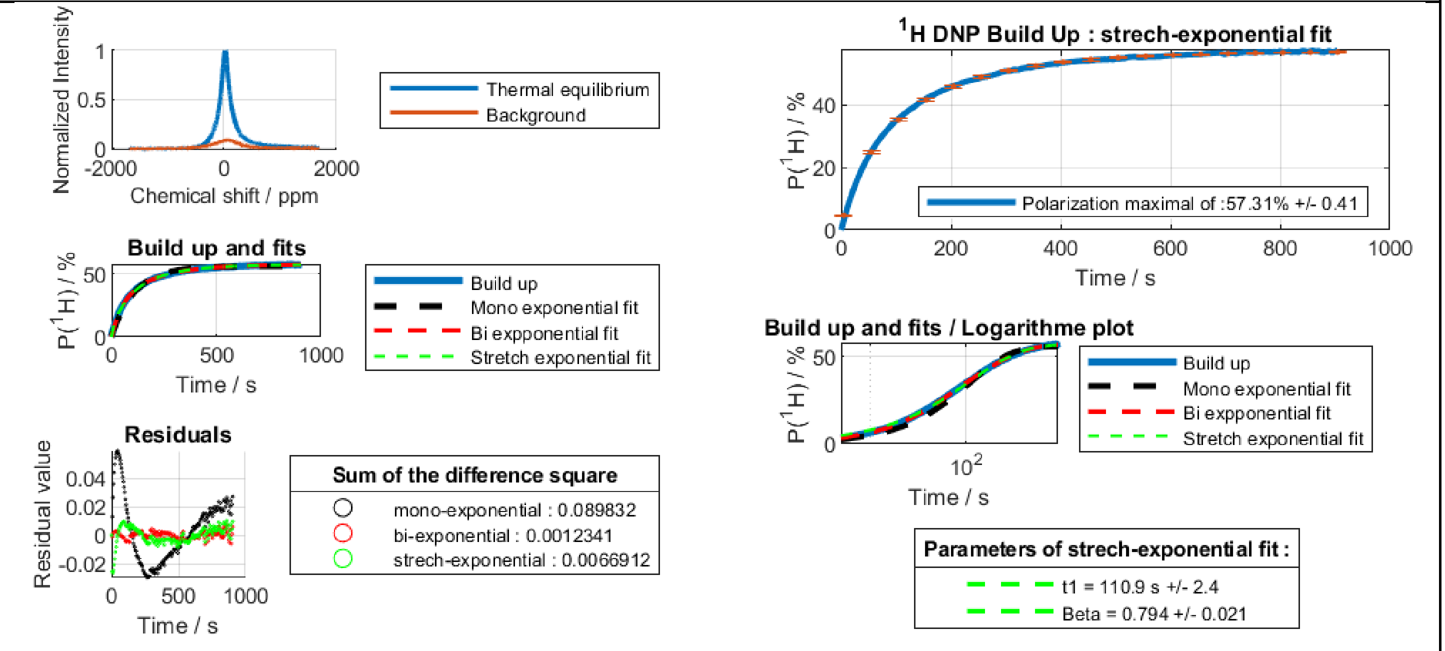

TED 65 / 116 µmol. g-1

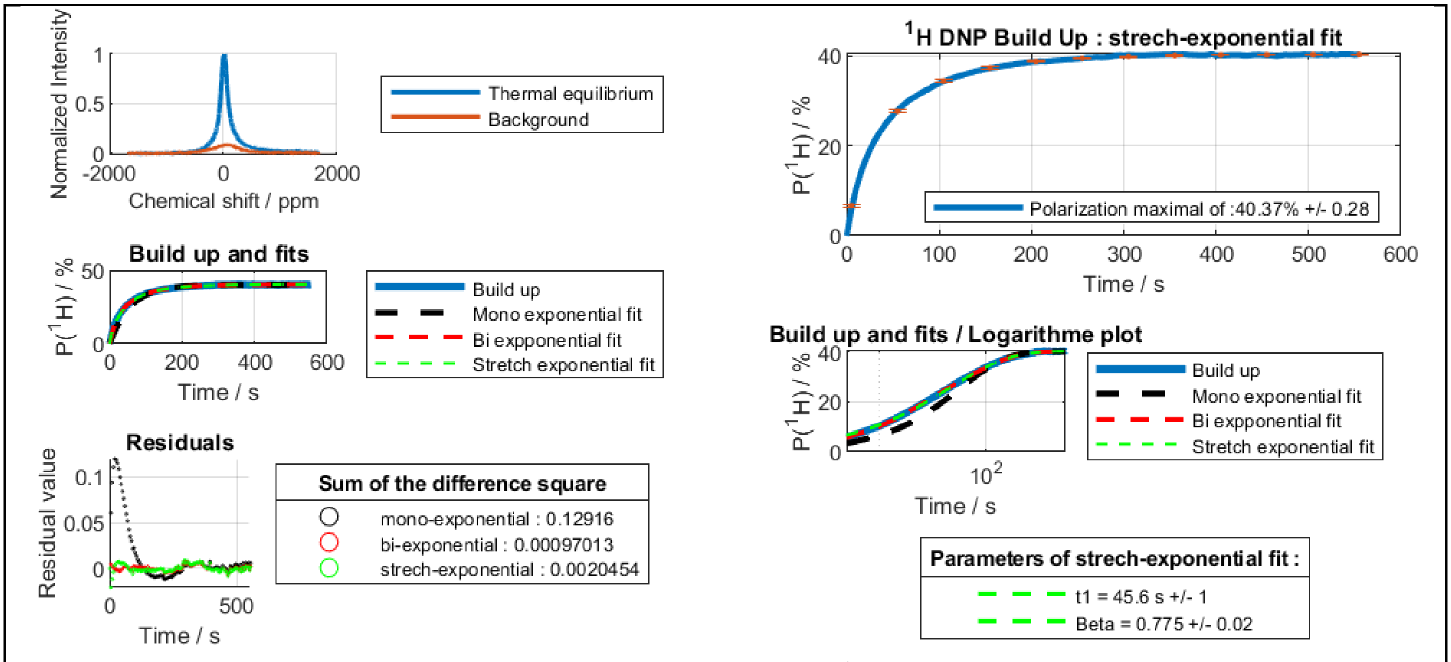

TED 66 / 192 µmol. g^{-1}

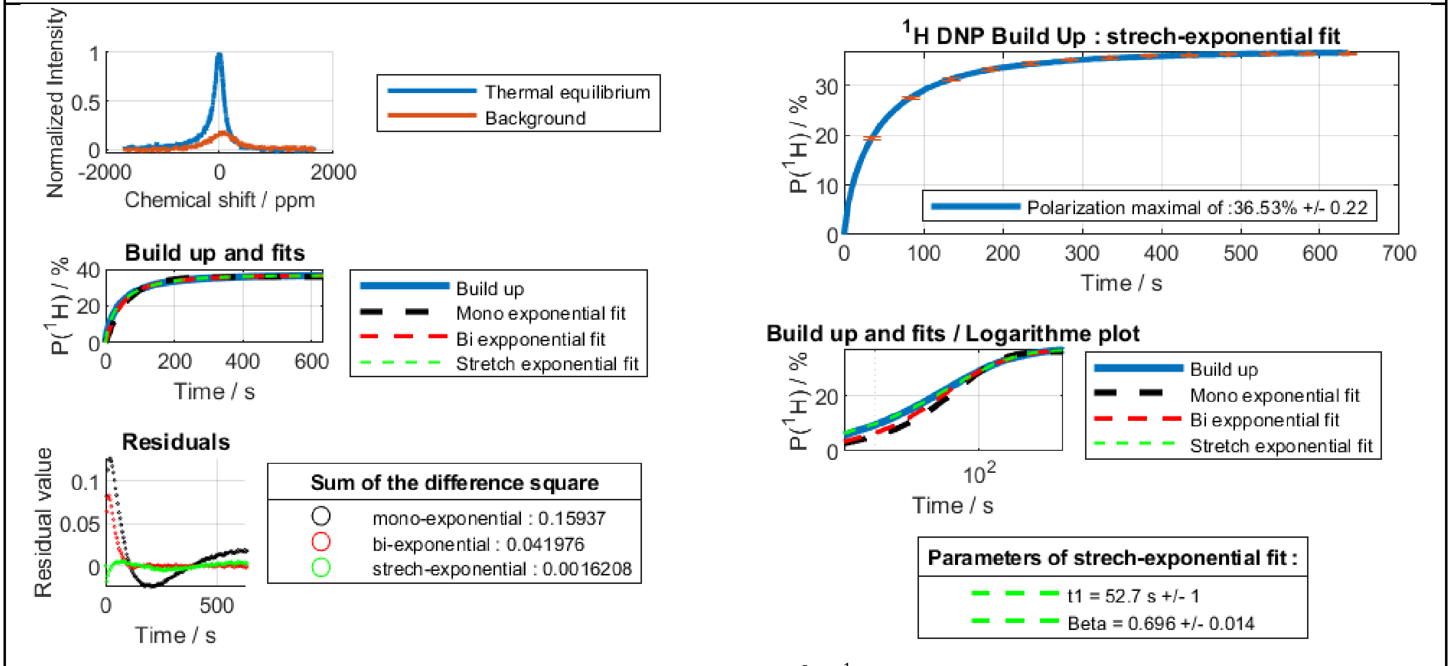

TED 67 / 285 µmol. g^{-1}

Supplementary Figure 10: *^{1}H DNP builds up.*

3 Supplementary References

1. Eaton GR, Eaton SS, Barr DP, Weber RT. *Quantitative EPR*. SpringerWi. Springer New York; 2010.
2. Huang H. Uncertainty estimation with a small number of measurements, part I: New insights on the t-interval method and its limitations. *Meas Sci Technol*. 2018;29(1).
3. Bornet A, Melzi R, Perez Linde AJ, et al. Boosting dissolution dynamic nuclear polarization by cross polarization. *J Phys Chem Lett*. 2013;4(1):111-114.
